# Not far enough: Public health policies to combat COVID-19 in Mexico’s states

**DOI:** 10.1371/journal.pone.0251722

**Published:** 2021-06-01

**Authors:** Felicia Knaul, Héctor Arreola-Ornelas, Thalia Porteny, Michael Touchton, Mariano Sánchez-Talanquer, Óscar Méndez, Salomón Chertorivski, Sonia Ortega, Mariana Chudnovsky, Pablo Kuri, the group from the Observatory for the Containment of COVID-19 in the Americas

**Affiliations:** 1 Institute for the Advances Study of the Americas, University of Miami, Miami, Florida, United States of America; 2 Leonard M. Miller School of Medicine, University of Miami, Miami, Florida, United States of America; 3 Tómatelo a Pecho, A.C., Ciudad de México, México; 4 Consejo Promotor de Universalidad y Competitividad en Salud, Fundación Mexicana para la Salud, A.C., Ciudad de México, México; 5 Research Center for Health Sciences, Anahuac University North Campus, Mexico City, Mexico; 6 Department of Community Health, Tufts University, Medford, MA, United States of America; 7 Department of Political Science, School of Arts and Sciences, University of Miami, Miami, Florida, United States of America; 8 Division of Political Science, Colegio de Mexico, Mexico City, Mexico; 9 Division of Public Administration, Center for Economic Research and Teaching (CIDE), Mexico City, Mexico; 10 Faculty of Medicine, National Autonomous University of Mexico, Mexico City, Mexico; University of Western Australia, AUSTRALIA

## Abstract

**Background:**

Mexican state governments’ actions are essential to control the COVID-19 pandemic within the country. However, the type, rigor and pace of implementation of public policies have varied considerably between states. Little is known about the subnational (state) variation policy response to the COVID-19 pandemic in Mexico.

**Material and methods:**

We collected daily information on public policies designed to inform the public, as well as to promote distancing, and mask use. The policies analyzed were: School Closure, Workplace Closure, Cancellation of Public Events, Restrictions on Gatherings, Stay at Home Order, Public Transit Suspensions, Information Campaigns, Internal Travel Controls, International Travel Controls, Use of Face Masks We use these data to create a composite index to evaluate the adoption of these policies in the 32 states. We then assess the timeliness and rigor of the policies across the country, from the date of the first case, February 27, 2020.

**Results:**

The national average in the index during the 143 days of the pandemic was 41.1 out of a possible 100 points on our index. Nuevo León achieved the highest performance (50.4); San Luis Potosí the lowest (34.1). The differential between the highest versus the lowest performance was 47.4%.

**Conclusions:**

The study identifies variability and heterogeneity in how and when Mexican states implemented policies to contain COVID-19. We demonstrate the absence of a uniform national response and widely varying stringency of state responses. We also show how these responses are not based on testing and do not reflect the local burden of disease. National health system stewardship and a coordinated, timely, rigorous response to the pandemic did not occur in Mexico but is desirable to contain COVID-19.

## Introduction

Latin America became a global epicenter of SARS-CoV2 virus infections and deaths from its associated disease, COVID-19, over the summer of 2020. The pandemic that started in Wuhan, China, at the beginning of 2020, quickly passed to Europe, Canada and the USA [1–3]. Despite the fact that Latin America only has 8% of the global population, since the last week of May it has consistently contributed more than 40% of the daily deaths in the world [4, 5]. Mexico has become an epicenter within the epicenter [6–8] with total accumulated deaths and daily deaths at levels that are highly disproportionate to its population [6–8]. Mexico accounts for roughly 20% of Latin America’s population. The current total deaths in Mexico due to COVID-19 (approximately 320,000), is roughly 40% of the regional total of ~723,000, which is disproportionate to Mexico’s population [9]. The federal response has been limited in each country, placing the public health policymaking burden on the states. The states have responded in widely distinct ways, with substantial variability in policies as well as in the number of cases, deaths and public health responses from authorities [10]. The result in Mexico is 32 distinct pandemics, not one, and important lessons for countries across the region and around the world.

Mexico is a federal country comprised of 32 partially self-governing states. Legal regulations grant states broad responsibilities as health authorities within the National Health System. These responsibilities include containing epidemics. As stated in the General Health Law [11], state governments have the obligation to implement health security measures according to the magnitude of the epidemic and establish mechanisms to reduce the mobility of inhabitants within its borders. This important role has been reinforced throughout the COVID-19 pandemic. When the national government declared the country to be in phase 3 on April 21, the Ministry of Health ratified it through an agreement published in the Official Gazette of the Federation. The agreement gives state governments the responsibility to implement the necessary and adequate public policies to achieve the physical distancing of the population [12].

Mexican state governments’ actions are essential to control the pandemic within the country [13]. However, the type, rigor and pace of implementation of public policies have varied considerably between states, despite the fact that the World Health Organization (WHO) and the international scientific community recommended implementing immediate hygiene and physical distancing measures to reduce the speed of contagion [14–18]. In turn, we expect that the differences in states’ implementation of health measures to control COVID-19 will have a direct and important impact on the health of the Mexican population, as well as on the possibility of controlling the pandemic at the national level. Moreover, the heterogeneity in the governmental response between the states of the country has occurred against the background of pre-existing territorial and social inequalities in the coverage and quality of health services [19, 20]. The result will likely be broad heterogeneity in health outcomes across the Mexican states with some states far outperforming others in containing the pandemic, treating infected citizens, and supporting their recovery.

**Fig 1:**
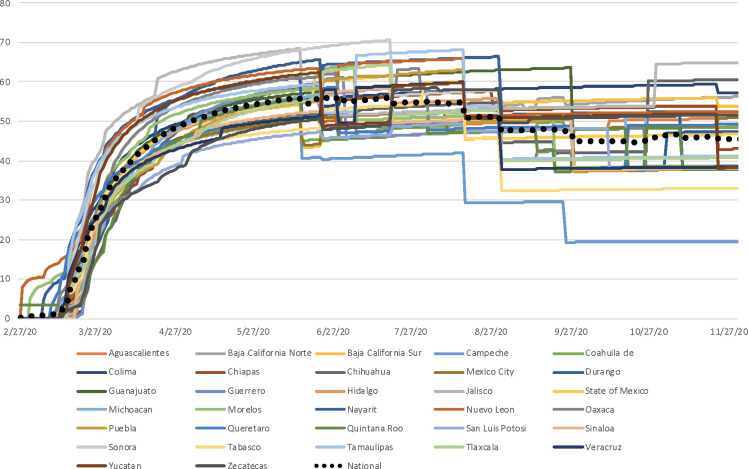
Public policy index for the containment of COVID-19 in Mexico. From February 27, 2020 to November 30, 2020.

In Mexico, federal measures to institute physical distancing or the so-called “National Healthy Distance Day” began on March 23, 2020, more than three weeks after the first recorded case in the country. On March 14^th^, public education authorities announced that activities were suspended beginning on the 20th of the same month. On March 24, the official beginning of phase 2, “community transmission”, was declared at the national level, thus suspending non-essential government activities and reinforcing confinement measures. During the months of March, April, May and the first weeks of June, 2020, the national health authorities did not recommend the use of face masks for the general population, despite the evidence suggesting that their use is effective in mitigating contagion [2, 15, 21–23]. Thus, compared to most Latin American countries, Mexico lagged behind in the application of national measures of physical distancing and containment of the epidemic, starting from the date of the first detected case of COVID-19. However, the Mexican states each reacted differently relative to the federal response. Fig 1 in S1 Appendix presents Mexico’s national policy response over the course of the pandemic compared to seven other Latin American countries for the indicators we collect.

The purpose of this paper is to present an analysis of the state-level variation in the public health response to the epidemic in Mexico. The analysis is drawn from a unique, daily database of ten public policy measures and how they were implemented sub-nationally. These measures are: 1. School closings; 2. Working from home for non-essential workers, 3. Cancellation of public events, 4. Suspension of public transport, 5. The development of information campaigns, 6. Restriction of trips within the state, 7. Control of international trips, 8. Stay at home guidelines, 9. Restrictions on the size of gatherings and 10. Guidelines for mask coverage.

Our analysis is comprised of a three-step process. First, we analyze each of these 10 policies for each state. Second, we build a composite index, taking data from February 27 (the first case reported in the country) to July 19, 2020. Third, we describe the heterogeneity between the states, in terms of the efficacy and promptness of their response to contain population mobility and promote the use of public health measures among the population.

## Materials and methods

### Public health policy variables

We developed an ecological study design, with data collection beginning in April 2020 and extending backward to the first recorded case in the country. The study extends to November 30^th^, 2020. We analyzed 10 variables that are part of the array of policies to contain SARS-CoV2 in each of the 32 Mexican states. Daily data on these policies begins on February 27^th^, which corresponds to the date of the first case reported in Mexico up until November 30th. We focus on indicators specific to the mobility restrictions and containment of the virus, as these can help explain the health impact in terms of the cases and deaths brought by COVID-19.

We examined whether each measure was being implemented each day, from the date of the first case detected in the country. If it was, we ascertained how rigorously the policy was implemented by coding its application as partial or full. To ensure the quality of the data, a double-blind review was carried out between two of the members of the group. In cases of discrepancy, the whole working group deliberated on the coding until consensus was reached. The breakdown of sources by entity is presented in the methodological appendix (Table 1A in S1 Appendix). Finally, we weigh the timeliness of each of these measures, determined by the date of their adoption.

Table 1 describes the 10 variables and the possible values in their measurement. We assign several discrete levels to the variables to achieve greater granularity in the analysis. These values were determined through deliberations with the research team and advice from external scientists such as the OxCGRT COVID policy tracking team at Oxford University. The variables "school closings", "work from home", "cancellation of public events", "suspension of public transport and / or closure of public transport systems" are categorical and take values of 0 when they have not been implemented, 0.5 when implementation is partial and 1 when it is total. The variable "development of information campaigns" is evaluated through the relative presence or absence of an informational strategy about the virus, the disease, its consequences and containment measures. Values of 0 are assigned if there were no campaigns; 0.5 represents the existence of campaigns only of a federal nature; and 1 when there is a state campaign. The variable "travel restrictions within the state" records the implementation of restrictions on internal movement in the state and takes values of 0 when they are not applied; 0.5 when restriction of movement is recommended; and 1 when the state restricted internal movement.

**Table 1:** Public policy indicators to contain COVID-19.

Identifier	Name	Description	Coding
I_1_		Record of School and University Closures	0: No Closure;
	School Closure		0.5: Partial Closure;
			1: Complete Closure
I_2_		Record of Work-Place Closures	0: No Closure;
	Workplace Closure		0.5 = Partial Business Closure
			1 = Complete Closure
I_3_		Record of the Cancellation of Public Events	0: No Closure;
	Cancellation of Public Events		0.5: Partial Closure;
			1: Complete Closure
I_4_		Record of Legal Restrictions on Private Gatherings	0: No Restrictions;
			0.25: Bans on Gatherings of More than 1000 People;
	Restrictions on Gatherings		0.5: Bans Restricting Gatherings between 100 and 1000;
			0.75: Bans Restricting Gatherings between 50 and 100;
			1: Bans on Gatherings of More than 10 People
I_5_		Record of "Shelter in Place" and other Orders Instructing Individuals to Stay at Home	0: No Order;
	Stay at Home Order		0.5: Partial Order;
			1: Full Order
I_6_		Record of Suspension of Public Transit	0: No Closure;
	Public Transit Suspensions		0.5: Partial Closure;
			1: Full Closure
I_7_		Record of Public Information/Health Campaigns	0: No Campaign;
	Information Campaigns		0.5: Very Limited Campaign
			1: Full Campaign
I_8_		Record of Restrictions on Internal Travel	0: No Closure;
	Internal Travel Controls		0.5: Partial Closure;
			1: Full Closure
I_9_		Record of Restrictions on International Travel	0: No Closure;
	International Travel Controls		0.5: Partial Closure;
			1: Full Closure
I_10_		Record of Mask Mandates	0: No Masks Required
	Use of Face Masks		0.5: Masks Recommended
			1: Masks Required in Public

The variable "international travel control guidelines" records international movement restrictions, taking values of 0 when no action was taken; 0.33 when only screening and/or monitoring is applied to international travelers; 0.66 when mandatory quarantine is ordered for travelers in high-risk regions; and 1 when the travel ban to and from high-risk regions is implemented. However, some states may not respond adequately to this variable because they do not have an international sea or airport; Consequently, giving a value of 0 to the states that do not have borders, ocean ports or airports would penalize the state unfairly. For this reason, states without forms of international travel were assigned the value of the daily national average, which corresponds to the states that did respond to said public policy. The stay-at-home guidelines variable measures orders to shelter or confine oneself to the home and takes values of 0 when no recommendation has been issued; 0.33 when there is a recommendation not to leave the house; 0.66 when the instruction is not to leave home except in "essential" cases; and 1 when the closure is complete or requires not leaving the home with minimal exceptions. The variable “restrictions on the size of meetings” refers to the cut-off size on the prohibitions of private meetings, taking values of 0 in the absence of any indication in this regard; 0.25 when the restriction is for meetings of more than 1,000 people; 0.5 applies when the meetings are between 100 and 1,000 people; 0.75 to meetings between 10 and 100; and 1 to meetings of less than 10 people. Table 1 presents the coding for each indicator included in our public policy index. Table 3 presents the mean and standard deviation for each indicator, by state, for the duration of the timeframe under investigation.

As of April 6, the WHO recommended the use of face masks. Therefore, from that date on, we retrospectively added a variable to describe its implementation in each state [24, 25]. This variable takes values of 0 if there are no guidelines; 0.5 when there is a recommendation to use masks; and 1 when mask use is mandatory.

### Public policy adoption index

We generated an index that combines the ten variables to create a summary view of state governments’ actions and allows for direct comparisons of how they inform the public, restrict population mobility, maintain public safety, and manage the economic re-opening.

The index is constructed as presented in the following Eq 1:

$$IPP_{i_{t}}=\left\{\sum_{j=1}^{n}I_{j_{t}}*[{\left(\frac{d_{j_{t}}}{D_{t}}\right)}^{\wedge }(\frac{1}{2})]/10\right\}*100$$ (1)

         Whereby:

   ${IPP}_{i_{t}}$ = Public policy adoption index in country/state *i* in time *t*.

      *Ij* = Public Policy Index *j*, where *j* goes from 1 to n = 10.

      *Dt* = Days from the first registered case until time *t*.

      *dt* = Days from the implementation of policy *j* until time *t*.

The ${IPP}_{i_{t}}$ is constructed with the sum of each of the values from the 10 variables, weighted by the day of implementation of each one in relation to the appearance of the first case; the index gives greater weight to early implementation relative to the first case in the country. As such, the index acquires higher values the earlier a certain measure has been implemented.

The ratio *dt / Dt* is continuous and goes from 0, when policy *j* has not yet been implemented in state *i* at time *t*, up to 1, in instances where public policy has been implemented at the same time *t* in which the first case appears. This makes it possible to take into account that public containment policies have less effect on containing the virus the later they are adopted. To this end, we raise the ratio *dt / Dt* to the power (1/2), to reflect decreasing policy efficacy with delays in policy implementation. For more detail, please review the methodological S1 Appendix.

In the aggregate, each state *i* receives a daily score between 0 and 10, which reflects the sum of the different policy dimensions and then normalized to 100. The maximum value of the index is 100 but obtaining it would not be realistic or desirable since it would imply a total closure of the state the day after the first case.

### Sources of information

We gathered data from three types of publicly available sources. First, we reviewed official government websites and state registers for each of the 32 states and the federal district, to capture laws, decrees, and news items specifying implementation of each public policy variable. Then, we cross-referenced this material against multiple news’ outlets’ database of Mexican state laws and decrees. We also used official newspapers, local newspapers and news shared by representatives on social media accounts such as Twitter Finally. See Table 1A in S1 Appendix for the breakdown of sources by entity. The data that we present in this article are from February 26 to November 30th, 2020.

A double-blind review was carried out by two of the authors to ensure the quality of the data. The review first consisted of randomly selecting members of the group to review randomly selected scores from among those that others coded. Next, these coders re-coded data for those states without having seen the original scores. The second coder did not know who coded the original data and the original coder did not know who would do the review. In cases of discrepancy, the whole working group deliberated on the coding until consensus was reached.

## Results

Table 2 presents the main sociodemographic statistics by state [26, 27].

**Table 2:** Main sociodemographic statistics by state.

State	Population (2020)	Marginalization Index (2015)	Level of marginalization (2015)	GDP Per Capita (2018)	% Population Below the Poverty Line (2018)	Public spending on health (as % of GDP) (2018)	Public Health Spending Per Capita (2018)
Aguascalientes	1,434,635	-0.89	Low	218,086	26.2	2.4	5,633
Baja California	3,634,868	-1.1	Very Low	204,619	23.3	2.5	5,065
Baja California Sur	804,708	-0.6	Low	289,263	18.1	2.3	6,524
Campeche	1,000,617	0.46	High	549,456	46.2	1.0	6,020
Coahuila	5,730,367	2.41	Very High	145,958	22.5	2.0	5,381
Colima	3,801,487	-0.6	Low	36,546	30.9	3.2	5,841
Chiapas	3,218,720	-1.1	Very Low	105,820	76.4	5.3	3,294
Chihuahua	785,153	-0.73	Low	955,086	26.3	2.8	5,575
Mexico City	9,053,990	-1.45	Very Low	401,060	30.6	2.9	11,947
Durango	1,868,996	0.05	Medium	137,777	37.3	3.4	4,863
Guanajuato	6,228,175	-0.07	Medium	157,075	43.4	2.8	4,538
Guerrero	3,657,048	2.56	Very High	83,837	66.5	4.9	4,181
Hidalgo	3,086,414	0.5	High	120,988	43.8	3.2	4,056
Jalisco	8,409,693	-0.82	Low	187,299	28.4	2.4	4,646
State of Mexico	17,427,790	-0.57	Low	112,403	42.7	3.8	4,233
Michoacan	4,825,401	0.5	High	115,901	46.0	3.2	3,821
Morelos	2,044,058	-0.2	Medium	121,557	50.8	3.5	4,396
Nayarit	1,288,571	0.31	Medium	119,990	34.8	4.0	4,779
Nuevo Leon	5,610,153	-1.39	Very Low	302,258	14.5	1.6	5,033
Oaxaca	4,143,593	2.12	Very High	84,957	66.4	4.6	3,943
Puebla	6,889,660	0.69	High	110,751	58.9	3.1	3,709
Queretaro	2,279,637	-0.49	Low	231,246	27.6	1.9	4,734
Quintana Roo	1,723,259	-0.37	Medium	205,234	27.6	2.4	4,918
San Luis Potosi	2,866,142	0.58	High	175,802	43.4	2.3	4,033
Sinaloa	3,156,674	-0.24	Medium	155,199	30.9	2.9	4,681
Sonora	3,074,745	-0.7	Low	243,736	28.2	2.5	6,013
Tabasco	2,572,287	0.3	Medium	191,878	53.6	2.6	5,234
Tamaulipas	3,650,602	-0.62	Low	178,564	35.1	3.1	5,563
Tlaxcala	1,380,011	-0.2	Medium	91,855	48.4	4.3	4,140
Veracruz	8,539,862	1.14	High	117,845	61.8	3.8	4,606
Yucatan	2,259,098	0.51	High	144,795	40.8	4.3	6,405
Zacatecas	1,666,426	0.01	Medium	121,356	46.8	3.8	4,729
National	128,112,840	-0.02	Medium	173,216	41.9	2.8	5,223

/1 Consejo Nacional de Población (CONAPO). (2019). “Cuadernillos Estatales de las Proyecciones de la Población de México y de las Entidades Federativas, 2016–2050. Proyecciones de la Población en México y de las Entidades Federativas”. Recuperado el 26 de junio de 2020 de https://www.gob.mx/conapo/documentos/cuadernillos-estatales-de-las-proyecciones-de-la-poblacion-de-mexico-y-de-las-entidades-federativas-2016-2050-208243?idiom=es

/2 Consejo Nacional de Población (CONAPO). (2016). “Índice de marginación por entidad federativa y municipio 2015”. Recuperado el 26 de junio de 2020 de https://www.gob.mx/conapo/documentos/indice-de-marginacion-por-entidad-federativa-y-municipio-2015

3/ Instituto Nacional de Estadística y Geografía (INEGI). (2019). “PIB por Entidad Federativa (PIBE). Base 2013”. Recuperado el 26 de junio de 2020 de https://www.inegi.org.mx/programas/pibent/2013/

4/ Consejo Nacional de Evaluación de la Política de Desarrollo Social (CONEVAL). (s.f). “Pobreza en México. Resultados de pobreza en México 2018 a nivel nacional y por entidades federativas”. Recuperado el 26 de junio de 2020 de https://www.coneval.org.mx/Medicion/MP/Paginas/Pobreza-2018.aspx

5/ Dirección General de Información en Salud—Secretaría de Salud (DGIS-SS)). (s.f). “Recursos en Salud. Cubos Dinámicos”. Recuperado el 26 de junio de 2020 de http://www.dgis.salud.gob.mx/contenidos/basesdedatos/bdc_recursos_gobmx.html

Table 3 presents descriptive statistics of the 10 public policy variables, up to November 30, 2020. Results indicate that “School closing” was the most homogeneously implemented policy. The national weighted average for this variable is 0.92, with 255 days of implementation. However, the length of time the policy has been in place ranges from 243 days of implementation in Puebla, to 200 days in Querétaro and Tlaxcala.

**Table 3:** Descriptive statistics of the 10 public policy variables by state. From February 27 to November 30, 2020.

		School closing	Workplace closing	Cancel public events	Close public transport	Public information campaigns	Restrictions on internal movement	International travel restrictions	Stay at home measures	Restrictions on sizes of gatherings	Mask-wearing guidelines
**Aguascalientes**	Mean	0.92	0.40	0.66	0.14	0.91	0.00	0.00	0.68	0.66	0.82
	Std. Dev.	0.27	0.16	0.28	0.23	0.29	0.00	0.00	0.39	0.24	0.36
	Days of implementation	256	252	263	248	253	0	0	253	246	237
**Baja California**	Mean	0.92	0.36	0.94	0.00	0.91	0.21	0.19	0.68	0.66	0.41
	Std. Dev.	0.27	0.15	0.25	0.00	0.29	0.25	0.24	0.39	0.24	0.19
	Days of implementation	249	245	253	0	246	242	106	243	239	221
**Baja California Sur**	Mean	0.92	0.40	0.83	0.00	0.91	0.13	0.17	0.68	0.66	0.77
	Std. Dev.	0.27	0.16	0.30	0.00	0.29	0.22	0.24	0.39	0.24	0.42
	Days of implementation	256	252	258	0	253	244	262	253	246	213
**Campeche**	Mean	0.92	0.21	0.58	0.00	0.46	0.16	0.16	0.40	0.45	0.81
	Std. Dev.	0.26	0.22	0.34	0.00	0.14	0.23	0.23	0.36	0.34	0.39
	Days of implementation	256	246	264	0	253	259	257	252	246	225
**Coahuila**	Mean	0.92	0.36	0.63	0.00	0.90	0.12	0.00	0.73	0.67	0.79
	Std. Dev.	0.27	0.15	0.30	0.00	0.30	0.21	0.00	0.35	0.24	0.41
	Days of implementation	256	252	257	0	251	235	0	252	247	220
**Colima**	Mean	0.93	0.45	0.93	0.17	0.93	0.32	0.00	0.68	0.66	0.40
	Std. Dev.	0.25	0.13	0.25	0.24	0.25	0.15	0.00	0.38	0.24	0.20
	Days of implementation	259	259	259	256	259	242	0	254	246	225
**Chiapas**	Mean	0.92	0.33	0.94	0.26	0.87	0.00	0.00	0.73	0.64	0.238
	Std. Dev.	0.27	0.16	0.24	0.25	0.30	0.00	0.00	0.35	0.26	0.12
	Days of implementation	256	252	261	241	256	0	0	250	246	221
**Chihuahua**	Mean	0.91	0.31	0.64	0.12	0.86	0.20	0.46	0.63	0.67	0.82
	Std. Dev.	0.29	0.20	0.36	0.21	0.34	0.21	0.14	0.32	0.22	0.38
	Days of implementation	253	252	251	226	240	249	256	253	252	231
**State of Mexico**	Mean	0.92	0.33	0.73	0.13	0.91	0.00	0.00	0.68	0.69	0.8345
	Std. Dev.	0.27	0.15	0.31	0.22	0.29	0.00	0.00	0.39	0.19	0.36
	Days of implementation	256	246	259	241	253	0	0	253	261	236
**Durango**	Mean	0.91	0.29	0.54	0.19	0.93	0.30	0.16	0.73	0.66	0.82
	Std. Dev.	0.29	0.18	0.34	0.23	0.26	0.18	0.23	0.34	0.25	0.38
	Days of implementation	253	252	243	250	258	251	251	255	243	229
**Guanajuato**	Mean	0.93	0.27	0.76	0.32	0.93	0.31	0.00	0.59	0.68	0.42
	Std. Dev.	0.25	0.20	0.36	0.24	0.25	0.24	0.00	0.33	0.20	0.19
	Days of implementation	259	252	259	243	259	235	0	247	259	232
**Guerrero**	Mean	0.91	0.34	0.57	0.11	0.91	0.16	0.15	0.57	0.67	0.41
	Std. Dev.	0.29	0.14	0.35	0.21	0.29	0.23	0.23	0.28	0.22	0.19
	Days of implementation	253	252	252	215	253	242	244	255	252	228
**Hidalgo**	Mean	0.91	0.33	0.72	0.19	0.92	0.33	0.00	0.68	0.67	0.40
	Std. Dev.	0.29	0.15	0.33	0.24	0.28	0.16	0.00	0.38	0.22	0.20
	Days of implementation	253	252	252	255	255	243	0	259	252	221
**Jalisco**	Mean	0.93	0.35	0.84	0.22	0.93	0.16	0.47	0.72	0.68	0.82
	Std. Dev.	0.25	0.13	0.28	0.25	0.25	0.23	0.13	0.33	0.19	0.38
	Days of implementation	259	252	263	259	259	259	252	256	263	228
**Mexico City**	Mean	0.91	0.34	0.69	0.19	0.93	0.33	0.00	0.50	0.68	0.83
	Std. Dev.	0.29	0.14	0.30	0.24	0.25	0.17	0.00	0.30	0.20	0.36
	Days of implementation	253	252	260	237	259	238	0	253	260	237
**Michoacan**	Mean	0.91	0.32	0.62	0.13	0.94	0.26	0.00	0.69	0.67	0.82
	Std. Dev.	0.29	0.18	0.31	0.22	0.25	0.18	0.00	0.32	0.22	0.38
	Days of implementation	252	251	260	231	260	226	0	260	252	229
**Morelos**	Mean	0.93	0.27	0.61	0.17	0.99	0.24	0.00	0.61	0.67	0.84
	Std. Dev.	0.26	0.21	0.34	0.24	0.12	0.21	0.00	0.29	0.22	0.37
	Days of implementation	258	252	258	244	274	235	0	264	252	234
**Nayarit**	Mean	0.91	0.36	0.75	0.30	0.97	0.362	0.16	0.66	0.67	0.64
	Std. Dev.	0.29	0.19	0.33	0.25	0.18	0.19	0.23	0.35	0.22	0.44
	Days of implementation	253	252	260	259	269	259	259	269	252	201
**Nuevo Leon**	Mean	0.91	0.37	0.71	0.28	1.00	0.36	0.17	0.73	0.68	0.86
	Std. Dev.	0.29	0.15	0.33	0.25	0.06	0.16	0.24	0.33	0.20	0.35
	Days of implementation	253	251	252	268	277	248	256	259	262	240
**Oaxaca**	Mean	0.91	0.32	0.63	0.20	0.90	0.20	0.20	0.59	0.67	0.81
	Std. Dev.	0.29	0.25	0.33	0.25	0.30	0.23	0.24	0.29	0.22	0.39
	Days of implementation	253	252	263	245	245	242	265	254	252	228
**Puebla**	Mean	0.91	0.30	0.67	0.21	0.93	0.25	0.00	0.62	0.67	0.831
	Std. Dev.	0.29	0.23	0.37	0.25	0.25	0.23	0.00	0.31	0.22	0.38
	Days of implementation	243	246	252	224	259	222	0	253	252	231
**Queretaro**	Mean	0.94	0.33	0.65	0.13	0.96	0.00	0.00	0.65	0.67	0.77
	Std. Dev.	0.25	0.17	0.30	0.22	0.20	0.00	0.00	0.36	0.22	0.39
	Days of implementation	260	252	254	228	266	0	0	264	252	229
**Quintana Roo**	Mean	0.91	0.30	0.56	0.15	0.86	0.15	0.00	0.67	0.67	0.8345
	Std. Dev.	0.29	0.19	0.33	0.23	0.35	0.23	0.00	0.30	0.22	0.37
	Days of implementation	253	253	245	242	239	252	0	278	252	232
**San Luis Potosi**	Mean	0.91	0.31	0.43	0.13	0.91	0.00	0.00	0.63	0.68	0.78
	Std. Dev.	0.29	0.20	0.14	0.22	0.29	0.00	0.00	0.33	0.20	0.40
	Days of implementation	253	253	260	231	253	0	0	253	259	224
**Sinaloa**	Mean	0.92	0.29	0.41	0.21	0.92	0.27	0.00	0.60	0.68	0.40
	Std. Dev.	0.27	0.21	0.15	0.25	0.27	0.22	0.00	0.33	0.21	0.20
	Days of implementation	256	252	256	252	256	241	0	253	256	223
**Sonora**	Mean	0.93	0.26	0.62	0.21	0.93	0.26	0.21	0.71	0.93	0.43
	Std. Dev.	0.25	0.23	0.36	0.25	0.25	0.23	0.25	0.33	0.25	0.17
	Days of implementation	259	253	259	248	259	255	211	253	259	241
**Tabasco**	Mean	0.91	0.28	0.43	0.26	0.47	0.28	0.00	0.58	0.68	0.81
	Std. Dev.	0.29	0.24	0.19	0.25	0.12	0.24	0.00	0.28	0.20	0.39
	Days of implementation	253	252	262	250	263	255	0	253	262	225
**Tamaulipas**	Mean	0.93	0.27	0.66	0.24	0.93	0.28	0.00	0.51	0.93	0.83453
	Std. Dev.	0.25	0.24	0.36	0.25	0.25	0.24	0.00	0.31	0.24	0.37
	Days of implementation	259	249	262	250	259	258	0	253	262	231
**Tlaxcala**	Mean	0.94	0.26	0.58	0.17	0.94	0.18	0.00	0.51	0.89	0.80
	Std. Dev.	0.25	0.23	0.37	0.24	0.25	0.22	0.00	0.31	0.31	0.40
	Days of implementation	260	252	248	226	260	208	0	253	248	222
**Veracruz**	Mean	0.93	0.27	0.41	0.24	0.94	0.26	0.00	0.51	0.65	0.43
	Std. Dev.	0.25	0.24	0.14	0.25	0.25	0.24	0.00	0.31	0.19	0.17
	Days of implementation	259	252	262	245	260	244	0	253	262	239
**Yucatan**	Mean	0.93	0.35	0.65	0.17	0.93	0.36	0.18	0.73	0.68	0.79
	Std. Dev.	0.25	0.16	0.33	0.24	0.25	0.15	0.24	0.33	0.21	0.40
	Days of implementation	259	252	249	255	259	259	262	253	253	221
**Zacatecas**	Mean	0.91	0.37	0.53	0.11	0.91	0.30	0.00	0.72	0.68	0.79
	Std. Dev.	0.29	0.15	0.24	0.21	0.26	0.19	0.00	0.36	0.22	0.41
	Days of implementation	252	252	254	224	261	212	0	253	254	220
**National**	Mean	**0.92**	**0.32**	**0.65**	**0.17**	**0.89**	**0.21**	**0.08**	**0.64**	**0.69**	**0.69**
	Std. Dev.	**0.27**	**0.18**	**0.30**	**0.21**	**0.25**	**0.18**	**0.08**	**0.34**	**0.23**	**0.33**
	Days of implementation	**255.06**	**251.38**	**256.56**	**212.28**	**257.06**	**204.84**	**90.03**	**255.19**	**253.09**	**227.31**

Maximum: Green

Minimum: Red

This measure was followed by “Public information campaigns” policy, with a national level of 0.89 and 257 days of implementation, and “mask-wearing guidelines”, with 0.69 and 227 days of implementation. The public policy with the lowest rate of implementation was “international travel restrictions”, which only reached a national level of 0.08, with 90 days of implementation. The Mexican federal government endorsed the use of facemasks on April 10^th^, later than other policies. There is substantial variation in its implementation. In this case, the values range from 0.24 in Chiapas, to 0.86 in Nuevo León, the state with the best score, followed by the Ciudad de México, Quintana Roo, and Tamaulipas with 0.84, and Puebla (83.1). The national average for this variable since the period the policy was implemented is 0.69. This is higher than restrictions on international travelers, travel restrictions within the state, suspension of public transport, and suspension of work, that some states implemented in the first days of March.

The second and third least implemented measures were "close public transport", with an average level of 0.17 and 212 days, and "restrictions on internal movement", with an average level of 0.21 and 205 days.

It should be noted that the correlation of each of the 10 individual public policy variables in the observed period (February 27 to November 30) was very high, ranging between 0.70 and 0.95. The use of face masks behaves differently from the others as expected (see methodological S1 Appendix). Please also see Table 2A in S1 Appendix for state-level data on total deaths, mortality rate, and fatality rate. See Table 3A in S1 Appendix of the Appendix for correlations between Covid-19 deaths and lagged policy index scores, by state, to assess the possibility that the burden of disease is driving policy choices among the states.

We analyze the performance of the states in the public policy index considering cuts at different dates (Table 4). As of June 14, some of these public policies began to relax with the implementation of the weekly epidemiological “traffic light”, approved by the General Health Council and put into effect by the federal government on June 1st. For this reason, we begin to see a drop in the index for some states. For example, until May 31 Jalisco had the highest score in the index; However, due to the relaxation of some policies, its average index fell and was surpassed by Nuevo León, with an average level of 50.4.

**Table 4:** Index scores for physical distancing policy adoption and containment of COVID-19 in Mexico by state. From February 27 to November 30, 2020.

	Until February 29th	Until March 31	Until April 30	Until May 31	Until June 30	Until July 31	Until August 31	Until September 30	Until October 31	Until November 30	Mean Index Score
Jalisco	0	47.35659	63.28271	67.33572	51.48935	52.44159	53.1777	53.59325	64.55663	64.84975	52.5969314
Nuevo León	9.072185	42.46935	57.95165	62.07162	64.30029	65.43747	51.55481	51.94167	52.24049	52.46473	52.2020939
Nayarit	0	43.53207	56.1614	60.02799	64.52032	65.6061	51.6276	52.00305	52.2933	38.50625	51.1981949
Colima	0	33.34629	49.8996	54.40194	56.10257	57.32844	58.15503	58.71015	59.13877	57.3154	50.0169675
Sonora	0	40.52565	59.30546	66.47271	69.48065	56.95437	40.26375	40.59581	40.85262	41.04552	48.6656318
Yucatán	0	39.75408	55.88987	60.66591	49.06281	59.25724	50.85899	51.34493	51.72027	38.2079	48.3334657
Tamaulipas	0	41.74946	54.12225	58.04123	52.28958	67.6606	40.38545	40.69994	40.94324	41.12603	47.0477256
Guanajuato	0	31.74693	49.12234	55.79036	60.48778	61.96772	62.95732	37.86012	38.11201	38.30112	46.8192419
Baja California	0	28.57295	44.46365	48.93438	57.39548	51.77824	53.84047	54.37518	55.47343	56.24206	46.1803971
Hidalgo	0	28.57644	48.04688	53.45245	60.51312	54.89345	50.60292	51.12647	51.53058	51.83369	46.0242238
Chiapas	0	24.26095	46.05963	50.77788	55.40645	56.78417	52.73238	53.26923	53.6836	42.98169	45.951444
Baja California Sur	0	29.9207	48.27785	53.05028	45.51111	46.63228	54.70133	55.27441	55.71672	53.9327	45.5349819
Chihuahua	0	21.16336	48.48602	56.62387	52.08973	53.88608	44.55789	41.97236	60.22984	60.63575	44.9436823
Morelos	0	34.44009	52.41871	56.88241	63.67218	50.92811	51.51813	38.30786	38.50219	38.64814	44.6178339
Mex	0	31.23183	48.60651	53.69579	60.50272	50.06751	45.63486	46.0743	46.41354	46.66803	44.4815523
Durango	0	23.46529	56.41195	63.33681	48.16432	49.43262	50.28422	37.32093	37.64406	47.38818	44.3516245
Oaxaca	0	34.13319	54.43876	59.80166	57.31033	58.34569	39.97269	37.30189	37.62736	37.87128	44.0129964
Michoacán	0	30.04423	48.76799	54.70344	48.80605	49.93997	50.70354	51.21428	51.60809	38.20869	43.8637906
Zacatecas	0	25.57504	38.72983	48.4697	48.19814	58.3015	49.60778	50.89433	51.33174	51.65904	43.7403971
Puebla	0	27.1209	45.98622	53.25208	60.82177	62.33445	49.94151	37.30579	37.63094	37.87458	43.3127437
Querétaro	0	34.32458	45.89191	49.9621	46.99399	49.82636	48.34954	38.12035	49.00067	49.21395	43.1388448
Ciudad de Mexico	0	29.03917	44.85911	49.19574	51.11796	52.22307	47.70546	48.16598	48.52164	50.87894	42.9718773
Aguascalientes	0	29.34663	44.80894	49.15416	45.63094	46.72678	49.52928	47.96297	50.44798	48.63232	42.6230325
Tlaxcala	0	27.66278	44.91277	53.27428	63.04856	51.89006	39.87054	40.2606	40.56172	40.78761	42.4697834
Coahuila	0	25.68668	44.87214	50.06896	45.37565	48.55472	47.30199	47.8217	48.22266	48.5233	41.8416968
Sinaloa	0	28.94434	44.61287	50.93661	57.5701	49.91502	50.67942	37.69625	37.96936	38.17435	41.8157762
Veracruz	0	28.83588	43.49974	49.8452	49.53286	59.4306	37.76046	38.0533	38.27985	38.45005	41.43787
Guerrero	0	25.84249	49.23035	57.27224	61.06485	46.6557	40.14325	40.57398	40.90642	41.15576	41.419639
Quintana Roo	3.333333	20.77391	45.92932	54.25241	45.73186	48.85763	47.55294	48.04057	48.41584	37.87507	41.0216245
San Luis Potosí	0	25.92071	41.24629	45.71997	45.91231	48.98218	47.64668	48.11561	37.90875	38.12064	39.0179422
Tabasco	0	31.43845	43.38514	47.01099	53.24112	54.34728	32.33595	32.61105	32.82368	32.98332	38.3294224
Campeche	0	30.36144	44.86697	50.68589	40.54442	41.54451	29.36573	19.32797	19.46322	19.56474	31.7449025
Nacional	0.4736417	31.48495	49.141	54.66989	55.29575	54.75908	47.58656	44.95203	46.44857	45.42369	44.8410181

Maximum: Green

Minimum: Red

To compare performance between states, we used the global average for the entire period as the indicator of the accumulated trend during the 277 days of the pandemic. Jalisco is the state with the highest average index (52.6), followed by Nuevo León (52.2), Nayarit (51.2), Colima (50.0), Sonora (48.7), Yucatán (48.3) and Tamaulipas (47.0). In contrast, we see the lowest scores in the index in the period in Campeche (31.7), followed by Tabasco (38.3), San Luis Potosí (39.0), Quintana Roo (41.0), Guerrero (41.4) and Veracruz (41.4). The national average in the period reached a level of 44.8. As shown, there are considerable differences in policy implementation across the country; the average index value for the state with the highest score is 65.7% greater than that of the lowest state.

Fig 1 provides a description of the timing and rigor in the adoption of policies in Mexican states in the first months of the pandemic. The graph reflects great heterogeneity in the timing of policy implementation to mitigate the spread of COVID-19. Some states, such as Nuevo León, Morelos, Nayarit, and Quintana Roo, anticipated federal instructions that devolved public health responsibility to the states and were the first to introduce policies to contain the virus. Others, such as Guerrero, Chihuahua, Sinaloa and Zacatecas, did not expect to be given this responsibility and acted later than the rest of the entities.

The graph also shows that states such as Sonora, Jalisco, and Nuevo León have implemented public policy measures with the greatest rigor. It is worth noting that the variance has increased during the period of time reported in this paper. This indicates that the difference in the number and rigor of public policies between states increased over time but began to converge as the national epidemiological traffic light came into effect.

Durango and Chihuahua, among others, adopted public health measures later than the other states, but showed improvement in the index towards the second and third week of April. For example, Chihuahua went from one of the worst states in the country to slightly above the national average in late May. In Durango, the policy correction was such that the state reached the ninth position (out of 32) in the average index as of June 12.

An additional group of states remained near the national average throughout the period and maintained regular policy implementation. These include the State of Mexico, Guanajuato, Tlaxcala, Puebla, Chihuahua, Michoacán, Colima, Querétaro, Guerrero, and Mexico City. Finally, some states have consistently underperformed in the index throughout the period, such as San Luis Potosí, Zacatecas, Chiapas, Coahuila, Baja California, Sinaloa, and Tabasco.

Finally, eighteen states had already begun to relax some of their policies, especially restrictions on public transportation, the suspension of work, and the directive to stay at home, during the latter portion of the timeframe under investigation. These relaxations are reflected in a considerable drop in policy index scores for Aguascalientes, Baja California Sur, Campeche, Chihuahua, Coahuila, Durango, Guerrero, Hidalgo, Jalisco, Michoacán, Morelos, Oaxaca, Querétaro, Quintana Roo, Tamaulipas, Veracruz, Yucatán, Zacatecas.

## Discussion

The governments of some states decided to implement public policies to combat COVID-19 before others and before national measures were implemented. Eight states of the republic—Guanajuato, Jalisco, Michoacán, Nuevo León, Tamaulipas, Tlaxcala, Veracruz and Yucatán—suspended classes and request their populations remain at home to avoid spreading the virus before the start of the National Healthy Distance Day. In this context, Nuevo León and Jalisco and its metropolitan areas of Monterrey and Guadalajara, respectively, stand out as positive examples. In both cases, state governments established policies to promote distancing, such as the cancellation of classes and mass events, before national measures were enacted. Other states, in contrast, limited themselves to following the guidelines of the federal government; these states reacted slowly and incompletely. Thus, both the number of public measures to contain contagion as well as their rigor and implementation time have varied considerably within the country during the health emergency.

In Mexico, the policy decisions of the state governments are essential to understand the evolution of the epidemic in the country. Given their legal powers, the state governments were the first line of defense in the face of the pandemic. Subnational governments acted at different times and with very different degrees of effectiveness. But, it is important to take into account the different socioeconomic circumstances facing the different states [28]. In some states, such as Quintana Roo, Baja California Sur, and Nayarit, whose main economic activity is tourism, governments implemented information campaigns and international travel restrictions.

State-level variation in terms of poverty is high in Mexico. Yet, we do not find a clear association between policy index scores and the states’ levels of poverty or marginalization. Chiapas, for example, has a similar level of poverty and marginalization to Oaxaca. Even though Chiapas’ average score improves in the latter dates of our reporting period, Chiapas displays a lower average performance than Oaxaca. Oaxaca demonstrates an average performance above the national value overall, but its index score fell in the last two weeks of the study due to the relaxation of its policies.

Our results point to the need for timely and rigorous state-level responses to contain the spread of infectious disease, particularly during a pandemic. Moving forward, it remains fundamental for states to be able to implement mitigation measures from the beginning of a pandemic, without having to wait for their state-powers to be ratified at the federal level. Our findings thus also show the need for clear mechanisms that guarantee states such ability and authority. Moreover, better coordination between states and multi-lateral organizations, like the Pan American Health Organization (PAHO) and the World Health Organization (WHO), is needed, especially in the absence of a consistent national response.

### Limitations

This work has some limitations. First, the analysis relies on a weighted index, based on the date of the first national case, suggesting that all states had to act at the same time. The index "penalizes" or "rewards" every state equally in terms of when policies began to be implemented. However, every state did not have its first case at precisely the same time. Second, the index weighs variables or policies as equally important in the containment of COVID-19. Yet, this is an assumption that requires empirical evaluation, once additional, accurate data on the number of cases, mortality, health services, case diagnosis, and mortality, become available. Third, the available data provides information on when policies were implemented but not on when decisions were made and the justifications that policymakers provided. Such information would be useful to better understand how decision-making processes impacted the course of the pandemic at the subnational level, which remains a goal for future research. Finally, we also acknowledge possible sources of bias from our sources, such as: vague language in state decrees, delays in posting decrees on government websites, failure to post decrees on websites, and failure to update websites when decrees were relaxed, abandoned, or re-implemented. As such, we maintained careful documentation of sources, cross-checked data, and documented all precedent-setting coding decisions to minimize such inconsistencies. Data included herein reflect the information available at the time of manuscript submission; new information is emerging rapidly during the pandemic, and states’ trajectories could shift over time.

## Conclusion

Our analysis shows how evaluating public policies at the national level hides important heterogeneity between states. This diversity of policy responses has a direct impact on the how effectively states contain the virus. The lack of policy uniformity for physical distancing and containment of COVID-19 in the country shows that state governments have been the main sources for policy in the area.

The heterogeneity in the states’ policy response highlights the need for a subnational approach to analyze government action to the COVID-19 pandemic–especially in the absence of a consistent national response. It is in this sense that the data and analysis presented here make an original and fundamental contribution [29, 30]. Other efforts to document, analyze and measure the effectiveness of the implementation of public policies in the face of COVID-19 have adopted a national vision [31], Those studies offer a useful overview, but are subject to what in the social sciences has been called the "whole country bias" [32]. Instead, we showcase the limitations of national-level, aggregate analyses by focusing on the subnational level in Mexico, a country with extensive territories and where subnational governments have played a crucial role to mitigate the spread of the pandemic.

A timely, rigorous, coordinated response to the pandemic has been missing in Mexico. The national government and many state governments have not gone far enough to implement NPI in a way that slows the spread of COVID-19.

## Supporting information

S1 Appendix (DOCX)

## References

[1] Liu Y., Gayle A.A., Wilder-Smith A., and Rocklöv J.: ‘The reproductive number of COVID-19 is higher compared to SARS coronavirus’, J Travel Med, 2020, 27, (2) 10.1093/jtm/taaa021 32052846 PMC7074654

[2] Mathur C.: ‘COVID-19 and India’s Trail of Tears’, Dialectical Anthropology, 2020, 44, (3), pp. 239–242 10.1007/s10624-020-09611-4 PMC7453091 32874013

[3] Munster V.J., Koopmans M., van Doremalen N., van Riel D., and de Wit E.: ‘A Novel Coronavirus Emerging in China—Key Questions for Impact Assessment’, N Engl J Med, 2020, 382, (8), pp. 692–694 10.1056/NEJMp2000929 31978293

[4] https://www.ft.com/content/a26fbf7e-48f8-11ea-aeb3-955839e06441, accessed 7/23/2020

[5] Dyer O.: ‘Covid-19 hot spots appear across Latin America’, Bmj, 2020, 369, pp. m2182 10.1136/bmj.m2182 32482681

[6] Rodriguez-Morales A.J., Gallego V., Escalera-Antezana J.P., Méndez C.A., Zambrano L.I., Franco-Paredes C., et al.: ‘COVID-19 in Latin America: The implications of the first confirmed case in Brazil’, in Editor (Ed.)^(Eds.): ‘Book COVID-19 in Latin America: The implications of the first confirmed case in Brazil’ (Elsevier USA, 2020, edn.), pp. 101613–101613 10.1016/j.tmaid.2020.101613 PMC7129040 32126292

[7] Lancet T.: ‘COVID-19 in Brazil:“So what?”‘, Lancet (London, England), 2020, 395, (10235), pp. 1461 10.1016/S0140-6736(20)31095-3 PMC7251993 32386576

[8] https://covid19.healthdata.org/mexico, accessed 7/23/2020

[9] ‘The Guardian. Mexico Covid death toll leaps 60% to reach 321,000. March 28th, 2020. Accessed on April 19th, 2020 https://www.theguardian.com/world/2021/mar/28/mexico-covid-death-toll-rise-60-percent

[10] Dong E., Du H., and Gardner L.: ‘An interactive web-based dashboard to track COVID-19 in real time’, The Lancet infectious diseases, 2020, 20, (5), pp. 533–534 10.1016/S1473-3099(20)30120-1 32087114 PMC7159018

[11] ‘Diario Oficial de la Federación. ACUERDO por el que se modifica el similar por el que se establecen acciones extraordinarias para atender la emergencia sanitaria generada por el virus SARS-CoV2’, in Editor (Ed.)^(Eds.): ‘Book Diario Oficial de la Federación. ACUERDO por el que se modifica el similar por el que se establecen acciones extraordinarias para atender la emergencia sanitaria generada por el virus SARS-CoV2’ (publicado el 31 de marzo de 2020, edn.), pp.

[12] Cruz Reyes, G., and Patiño Fierro, M.P.: ‘Las medidas del Gobierno Federal contra el virus SARS-CoV2 (COVID-19)’, 2020

[13] Ramírezde la Cruz E.E., Grin E.J., Sanabria‐Pulido P., Cravacuore D., and Orellana A.: ‘The Transaction Costs of the Governments’ Response to the COVID‐19 Emergency in Latin America’, Public Administration Review 10.1111/puar.13259 PMC7300791 32836458

[14] Nuguer, V., and Powell, A.: ‘2020 Latin American and Caribbean Macroeconomic Report: Policies to Fight the Pandemic’, in Editor (Ed.)^(Eds.): ‘Book 2020 Latin American and Caribbean Macroeconomic Report: Policies to Fight the Pandemic’ (2020, edn.), pp.

[15] Setti L., Passarini F., De Gennaro G., Barbieri P., Perrone M.G., Borelli M., et al.: ‘Airborne Transmission Route of COVID-19: Why 2 Meters/6 Feet of Inter-Personal Distance Could Not Be Enough’, Int J Environ Res Public Health, 2020, 17, (8) 10.3390/ijerph17082932 32340347 PMC7215485

[16] Wilder-Smith A., and Freedman D.O.: ‘Isolation, quarantine, social distancing and community containment: pivotal role for old-style public health measures in the novel coronavirus (2019-nCoV) outbreak’, Journal of travel medicine, 2020, 27, (2), pp. taaa020 10.1093/jtm/taaa020 32052841 PMC7107565

[17] Barrios J.M., Benmelech E., Hochberg Y.V., Sapienza P., and Zingales L.: ‘Civic Capital and Social Distancing during the Covid-19 Pandemic’, National Bureau of Economic Research Working Paper Series, 2020, No. 27320 10.1016/j.jpubeco.2020.104310 33199928 PMC7657101

[18] Munayco C.V., Tariq A., Rothenberg R., Soto-Cabezas G.G., Reyes M.F., Valle A., et al.: ‘Early transmission dynamics of COVID-19 in a southern hemisphere setting: Lima-Peru: February 29th–March 30th, 2020’, Infectious Disease Modelling, 2020, 5, pp. 338–345 10.1016/j.idm.2020.05.001 32399507 PMC7215155

[19] Cejudo G.M., Gómez-Álvarez D., Michel C.L., Lugo D., Trujillo H., Pimienta C., et al.: ‘Federalismo en COVID:¿ Cómo responden los gobiernos estatales a la pandemia?’

[20] Díaz de León-Martínez L., de la Sierra-de la Vega L., Palacios-Ramírez A., Rodriguez-Aguilar M., and Flores-Ramírez R.: ‘Critical review of social, environmental and health risk factors in the Mexican indigenous population and their capacity to respond to the COVID-19’, Science of the Total Environment, 2020, 733, pp. 139357–139357 10.1016/j.scitotenv.2020.139357 32416536 PMC7215151

[21] Leung N.H.L., Chu D.K.W., Shiu E.Y.C., Chan K.H., McDevitt J.J., Hau B.J.P., et al.: ‘Respiratory virus shedding in exhaled breath and efficacy of face masks’, Nat Med, 2020, 26, (5), pp. 676–680 10.1038/s41591-020-0843-2 32371934 PMC8238571

[22] Chu D.K., Akl E.A., Duda S., Solo K., Yaacoub S., and Schünemann H.J.: ‘Physical distancing, face masks, and eye protection to prevent person-to-person transmission of SARS-CoV-2 and COVID-19: a systematic review and meta-analysis’, Lancet, 2020 10.1016/S0140-6736(20)31142-9 PMC7263814 32497510

[23] Zhang R., Li Y., Zhang A.L., Wang Y., and Molina M.J.: ‘Identifying airborne transmission as the dominant route for the spread of COVID-19’, Proceedings of the National Academy of Sciences, 2020 10.1073/pnas.2009637117 PMC7334447 32527856

[24] World Health O.: ‘Advice on the use of masks in the context of COVID-19: interim guidance, 5 June 2020’, in Editor (Ed.)^(Eds.): ‘Book Advice on the use of masks in the context of COVID-19: interim guidance, 5 June 2020’ (World Health Organization, 2020, edn.), pp.

[25] Sanitaria, I. d.E.C.y.: ‘COVID-19. Información confiable para la toma de decisiones ‘, 2020

[26] Calvo, E., and Ventura, T.: ‘Will I get COVID-19? Partisanship, Social Media Frames, and Perceptions of Health Risk in Brazil *’, in Editor (Ed.)^(Eds.): ‘Book Will I get COVID-19? Partisanship, Social Media Frames, and Perceptions of Health Risk in Brazil *’ (2020, edn.), pp.

[27] Pulejo, M., and Querubín, P.: ‘Electoral Concerns Reduce Restrictive Measures During the COVID-19 Pandemic’, in Editor (Ed.)^(Eds.): ‘Book Electoral Concerns Reduce Restrictive Measures During the COVID-19 Pandemic’ (2020, edn.), pp. 10.1016/j.jpubeco.2021.104387 PMC7980214 33776156

[28] Ríos-Flores J.A., and Ocegueda Hernández J.M.: ‘Efectos de la capacidad innovadora en el crecimiento económico de las entidades federativas en México’, Estudios fronterizos, 2018, 19

[29] Luchini S., Teschl M., Pintus P., Baunez C., and Moatti J.P.: ‘Urgently Needed for Policy Guidance: An Operational Tool for Monitoring the COVID-19 Pandemic’, SSRN Electronic Journal, 2020

[30] Carrillo-Larco R.M.: ‘COVID-19 data sources in Latin America and the Caribbean’, in Editor (Ed.)^(Eds.): ‘Book COVID-19 data sources in Latin America and the Caribbean’ (Elsevier USA, 2020, edn.), pp. 10.1016/j.tmaid.2020.101750 PMC7258813 32479817

[31] González M.J.: ‘Características iniciales de las políticas de control de la pandemia de Covid-19 en América Latina’, Gac Méd Caracas 2020, 2020;128(2):207–21

[32] Snyder R.: ‘Scaling down: The subnational comparative method’, Studies in comparative international development, 2001, 36, (1), pp. 93–110

