# Supplementary material for: Not far enough: Public health policies to combat COVID-19 in Mexico’s states
Source: PLoS One. 2021 Jun 1;16(6):e0251722. doi: 10.1371/journal.pone.0251722 (PMC8168889; doi:10.1371/journal.pone.0251722)
Supplement: S1 Appendix — (DOCX) [file pone.0251722.s001.docx]

S1 Appendix

| **TABLE 1a. Sources of information for the 10 public health policies for the containment of COVID-19 in the different states in Mexico.** | | | | | | | |
| --- | --- | --- | --- | --- | --- | --- | --- |
|  |  |  |  |  |  |  |  |
|  | **School closures** | **Workplace closure** | **Canceling public events** | **Public transport closure** | **Information campaigns** | **Restrictions on gatherings** | **Restrictions on international travle** |
|  | Source | Source | Source | Source | Source | Source | Source |
| **Aguascalientes** | https://www.dof.gob.mx/nota_detalle.php?codigo=5589479&fecha=16/03/2020 | https://www.dof.gob.mx/nota_detalle.php?codigo=5590339&fecha=24/03/2020 | <https://eservicios2.aguascalientes.gob.mx/ssi/dnoticia.aspx?b=1762> |  | <https://www.aguascalientes.gob.mx/coronavirus/> |  |  |
|  |  | www.csg.gob.mx/descargas/pdf/index/informacion_relevante/COVID19_-_Presentacion_CSG_-_Medidas_Seguridad_Sanitaria.pdf | <https://www.dof.gob.mx/nota_detalle.php?codigo=5590339&fecha=24/03/2020> |  |  |  |  |
| **BCN** | https://www.dof.gob.mx/nota_detalle.php?codigo=5589479&fecha=16/03/2020 | https://www.dof.gob.mx/nota_detalle.php?codigo=5590339&fecha=24/03/2020 | <https://cadenanoticias.com/regional/2020/03/cancelan-eventos-masivos-en-bc> |  | [www.bajacalifornia.gob.mx/coronavirus](http://www.bajacalifornia.gob.mx/coronavirus) | <https://cadenanoticias.com/regional/2020/03/cierran-las-playas-de-rosarito-debido-al-covid-19> |  |
|  |  | www.csg.gob.mx/descargas/pdf/index/informacion_relevante/COVID19_-_Presentacion_CSG_-_Medidas_Seguridad_Sanitaria.pdf | <https://www.dof.gob.mx/nota_detalle.php?codigo=5590339&fecha=24/03/2020> |  |  |  |  |
| **BCS** | https://www.dof.gob.mx/nota_detalle.php?codigo=5589479&fecha=16/03/2020 | https://www.dof.gob.mx/nota_detalle.php?codigo=5590339&fecha=24/03/2020 | <https://www.milenio.com/estados/cierran-bares-cancelan-eventos-masivos-paz-comondu> |  | <https://coronavirus.bcs.gob.mx/> | <https://www.debate.com.mx/estados/Covid-19-cierra-Baja-California-Sur-hoteles-y-playas-por-emergencia-20200401-0087.html> | <https://www.elsudcaliforniano.com.mx/local/inicia-cancelacion-de-cruceros-a-baja-california-sur-coronavirusbcs-cruceroscoronavirus-covid19mexico-4969490.html> |
|  |  | www.csg.gob.mx/descargas/pdf/index/informacion_relevante/COVID19_-_Presentacion_CSG_-_Medidas_Seguridad_Sanitaria.pdf | <https://www.dof.gob.mx/nota_detalle.php?codigo=5590339&fecha=24/03/2020> |  |  |  |  |
| **Campeche** | https://www.dof.gob.mx/nota_detalle.php?codigo=5589479&fecha=16/03/2020 | https://www.dof.gob.mx/nota_detalle.php?codigo=5590339&fecha=24/03/2020 | [periodico.sfpcoahuila.gob.mx/ArchivosPO/22-EXT-19-MAR-2020.PDF](http://periodico.sfpcoahuila.gob.mx/ArchivosPO/22-EXT-19-MAR-2020.PDF) |  | <https://www.facebook.com/ucsgobcampeche/> | <https://www.poresto.net/2020/03/18/cierran-playa-bonita-en-campeche-por-coronavirus-covid-19/> | <https://oilandgasmagazine.com.mx/2020/04/marina-pone-en-cuarentena-a-buque-blue-pioneer-por-covid-19/> |
|  |  | www.csg.gob.mx/descargas/pdf/index/informacion_relevante/COVID19_-_Presentacion_CSG_-_Medidas_Seguridad_Sanitaria.pdf | <https://www.dof.gob.mx/nota_detalle.php?codigo=5590339&fecha=24/03/2020> |  |  |  |  |
| **Coahuila** | https://www.dof.gob.mx/nota_detalle.php?codigo=5589479&fecha=16/03/2020 | https://www.dof.gob.mx/nota_detalle.php?codigo=5590339&fecha=24/03/2020 | <https://www.excelsior.com.mx/nacional/estas-son-las-restricciones-que-aplica-coahuila-por-covid-19/1370647> |  | <http://www.saludcoahuila.gob.mx/COVID19/index.php> | <https://www.zocalo.com.mx/new_site/articulo/aislan-a-monclova-por-covid-19> |  |
|  |  | www.csg.gob.mx/descargas/pdf/index/informacion_relevante/COVID19_-_Presentacion_CSG_-_Medidas_Seguridad_Sanitaria.pdf | <https://www.dof.gob.mx/nota_detalle.php?codigo=5590339&fecha=24/03/2020> |  |  |  |  |
| **Colima** | http://www.periodicooficial.col.gob.mx/p/18032020/p20031801.pdf | http://www.periodicooficial.col.gob.mx/p/18032020/p20031801.pdf | http://www.periodicooficial.col.gob.mx/p/18032020/p20031801.pdf | https://diariodecolima.com/noticias/detalle/2020-03-20-por-contingencia-sanitaria-suspenden--ciertas-rutas-y-modifican-frecuencia-de-transporte-pblico | <http://saludcolima.gob.mx/coronavirus/> | <http://www.saludcolima.gob.mx/noticia.php?id=4534> |  |
|  |  | www.csg.gob.mx/descargas/pdf/index/informacion_relevante/COVID19_-_Presentacion_CSG_-_Medidas_Seguridad_Sanitaria.pdf | <https://www.dof.gob.mx/nota_detalle.php?codigo=5590339&fecha=24/03/2020> |  |  |  |  |
| **Chiapas** | https://www.dof.gob.mx/nota_detalle.php?codigo=5589479&fecha=16/03/2020 | https://www.dof.gob.mx/nota_detalle.php?codigo=5590339&fecha=24/03/2020 | <https://www.excelsior.com.mx/nacional/chiapas-cancela-eventos-masivos-por-covid-19/1369926> | <https://smyt.chiapas.gob.mx/> | <http://coronavirus.saludchiapas.gob.mx/> |  |  |
|  |  | www.csg.gob.mx/descargas/pdf/index/informacion_relevante/COVID19_-_Presentacion_CSG_-_Medidas_Seguridad_Sanitaria.pdf | <https://www.dof.gob.mx/nota_detalle.php?codigo=5590339&fecha=24/03/2020> |  |  |  |  |
| **Mexico City** | https://www.dof.gob.mx/nota_detalle.php?codigo=5589479&fecha=16/03/2020 | https://www.dof.gob.mx/nota_detalle.php?codigo=5590339&fecha=24/03/2020 | <https://www.eluniversal.com.mx/metropoli/cdmx/coronavirus-gobierno-de-cdmx-suspende-eventos-masivos-por-covid-19> | <https://politica.expansion.mx/cdmx/2020/04/02/el-metro-cdmx-anuncia-una-reduccion-escalonada-del-servicio-de-taquillas> | <https://www.eluniversal.com.mx/metropoli/cdmx/coronavirus-presentan-quedate-en-casa-campana-para-enfrentar-covid-19> |  |  |
|  |  | [www.csg.gob.mx/descargas/pdf/index/informacion_relevante/COVID19_-_Presentacion_CSG_-_Medidas_Seguridad_Sanitaria.pdf](http://www.csg.gob.mx/descargas/pdf/index/informacion_relevante/COVID19_-_Presentacion_CSG_-_Medidas_Seguridad_Sanitaria.pdf) | <http://data.consejeria.cdmx.gob.mx/portal_old/uploads/gacetas/887ed477612cfc31d077d94d4faa212f.pdf> | <https://semovi.cdmx.gob.mx/comunicacion/nota/tarjeta-informativa-cierre-temporal-de-estaciones-por-fase-3-covid-19> |  |  |  |
|  |  |  | <https://www.dof.gob.mx/nota_detalle.php?codigo=5590339&fecha=24/03/2020> |  |  |  |  |
| **Chihuahua** | <http://www.csg.gob.mx/descargas/pdf/index/informacion_relevante/COVID19_-_Presentacion_CSG_-_Medidas_Seguridad_Sanitaria.pdf> | <https://www.dof.gob.mx/nota_detalle.php?codigo=5590339&fecha=24/03/2020> | <http://www.cambio.gob.mx/spip.php?article14941> | <http://www.chihuahua.gob.mx/contenidos/nuevo-acuerdo-amplia-restricciones-y-suspension-de-actividades-no-esenciales-hasta-el-30> | <http://www.cambio.gob.mx/spip.php?article15072> | <https://entrelineas.com.mx/local/cierran-accesos-a-parral-contra-el-covid-19/> | <https://www.elheraldodechihuahua.com.mx/local/region/inicia-estados-unidos-cierre-de-frontera-con-mexico-noticias-de-chihuahua-4998239.html> |
|  |  | <http://www.csg.gob.mx/descargas/pdf/index/informacion_relevante/COVID19_-_Presentacion_CSG_-_Medidas_Seguridad_Sanitaria.pdf> |  |  |  | <https://www.proceso.com.mx/625370/toque-de-queda-y-cierre-de-municipios-de-chihuahua-por-temor-al-covid-19> |  |
| **Durango** | <http://www.csg.gob.mx/descargas/pdf/index/informacion_relevante/COVID19_-_Presentacion_CSG_-_Medidas_Seguridad_Sanitaria.pdf> | <https://www.dof.gob.mx/nota_detalle.php?codigo=5590339&fecha=24/03/2020> | <http://www.durango.gob.mx/se-acatara-en-durango-decreto-del-gobierno-federal/> | <https://www.elsoldedurango.com.mx/local/concesionarios-del-transporte-urbano-inician-paros-tecnicos-5019910.html> | <http://covid.durango.gob.mx/> | <https://www.elsiglodedurango.com.mx/noticia/1216625.por-covid-19,-cierran-entrada-a-municipio> | <https://lineadirectaportal.com/mexico/ultima-hora-cancelan-vuelos-en-aeropuertos-de-mexico-ante-emergencia-del-covid-19_20200325-979992/> |
|  |  | <http://www.csg.gob.mx/descargas/pdf/index/informacion_relevante/COVID19_-_Presentacion_CSG_-_Medidas_Seguridad_Sanitaria.pdf> |  |  |  |  |  |
| **Guanajuato** | <https://politica.expansion.mx/estados/2020/03/17/estados-adelantan-suspension-de-clases?_amp=true> | <https://www.dof.gob.mx/nota_detalle.php?codigo=5590339&fecha=24/03/2020> | <https://zonafranca.mx/cultura-y-entretenimiento/espectaculos/se-suspenden-conciertos-y-actividades-culturales-en-leon-por-coronavirus/> | <https://www.milenio.com/ciencia-y-salud/coronavirus-guanajuato-aplican-municipios-medidas-prevencion> | <https://coronavirus.guanajuato.gob.mx/> | <https://www.unotv.com/noticias/estados/guanajuato/detalle/coronavirus-en-penjamo-y-la-piedad-cierran-puentes-por-covid-19-984041/> | <https://salud.guanajuato.gob.mx/coronavirus.php> |
|  |  | <http://www.csg.gob.mx/descargas/pdf/index/informacion_relevante/COVID19_-_Presentacion_CSG_-_Medidas_Seguridad_Sanitaria.pdf> | <https://www.dof.gob.mx/nota_detalle.php?codigo=5590339&fecha=24/03/2020> |  |  |  |  |
| **Guerrero** | <http://www.csg.gob.mx/descargas/pdf/index/informacion_relevante/COVID19_-_Presentacion_CSG_-_Medidas_Seguridad_Sanitaria.pdf> | <https://www.dof.gob.mx/nota_detalle.php?codigo=5590339&fecha=24/03/2020> | <https://www.dof.gob.mx/nota_detalle.php?codigo=5590339&fecha=24/03/2020> | --- | <http://guerrero.gob.mx/articulos/recomendaciones-sobre-el-covid-19/> | <https://www.elnorte.com/aplicacioneslibre/preacceso/articulo/default.aspx?__rval=1&urlredirect=https://www.elnorte.com/cierran-pueblos-en-guerrero-por-covid-19/ar1911976?referer=--7d616165662f3a3a6262623b727a7a7279703b767a783a--> | <https://www.milenio.com/estados/guerrero-coronavirus-niegan-acceso-cruceros-acapulco> |
|  |  | <http://www.csg.gob.mx/descargas/pdf/index/informacion_relevante/COVID19_-_Presentacion_CSG_-_Medidas_Seguridad_Sanitaria.pdf> |  |  |  | <https://www.animalpolitico.com/2020/04/cierre-playas-acapulco-guerrerro-pandemia-covid-19/> |  |
| **Hidalgo** | <http://www.csg.gob.mx/descargas/pdf/index/informacion_relevante/COVID19_-_Presentacion_CSG_-_Medidas_Seguridad_Sanitaria.pdf> | <https://www.dof.gob.mx/nota_detalle.php?codigo=5590339&fecha=24/03/2020> | <https://www.dof.gob.mx/nota_detalle.php?codigo=5590339&fecha=24/03/2020> | <https://www.elsoldehidalgo.com.mx/local/transporte-publico-al-50-4998702.html> | <http://coronavirus.hidalgo.gob.mx/pag/MaterialConsulta.html#Cursos> | <https://www.eluniversal.com.mx/estados/coronavirus-pobladores-prohiben-la-entrada-comunidades-para-evitar-contagios-en-hidalgo> | --- |
|  |  | <http://www.csg.gob.mx/descargas/pdf/index/informacion_relevante/COVID19_-_Presentacion_CSG_-_Medidas_Seguridad_Sanitaria.pdf> |  |  |  |  |  |
| **Jalisco** | <https://politica.expansion.mx/estados/2020/03/17/estados-adelantan-suspension-de-clases?_amp=true> | <https://www.dof.gob.mx/nota_detalle.php?codigo=5590339&fecha=24/03/2020> | <https://www.jalisco.gob.mx/es/prensa/noticias/102580> | --- | <https://coronavirus.jalisco.gob.mx/que-es-el-covid-19/> | <https://www.eleconomista.com.mx/estados/Jalisco-ordena-el-cierre-de-playas-y-balnearios-por-la-contingencia-del-coronavirus--20200401-0069.html> | <https://www.radioformula.com.mx/noticias/20200324/vuelos-en-jalisco-suspendidos-contingencia-coronavirus-en-mexico/> |
|  |  | <http://www.csg.gob.mx/descargas/pdf/index/informacion_relevante/COVID19_-_Presentacion_CSG_-_Medidas_Seguridad_Sanitaria.pdf> |  |  |  |  |  |
| **Mexico** | <http://www.csg.gob.mx/descargas/pdf/index/informacion_relevante/COVID19_-_Presentacion_CSG_-_Medidas_Seguridad_Sanitaria.pdf> | <https://www.dof.gob.mx/nota_detalle.php?codigo=5590339&fecha=24/03/2020> | <https://www.gem.gob.mx/medios/w2detalle.aspx?tser=C&folio=12919> | <https://www.google.com.mx/amp/s/www.eluniversal.com.mx/metropoli/cancelan-servicio-de-transporte-de-toluca-santa-fe%3famp> | <http://salud.edomex.gob.mx/salud/covid#mas_informacion> | <https://www.elsoldetoluca.com.mx/local/tlatlaya-tambien-se-blinda-ante-contingencia-5072345.html> | --- |
|  |  | <http://www.csg.gob.mx/descargas/pdf/index/informacion_relevante/COVID19_-_Presentacion_CSG_-_Medidas_Seguridad_Sanitaria.pdf> |  | <https://www.gem.gob.mx/medios/w2detalle.aspx?tser=C&folio=13481> |  | <https://www.elgrafico.mx/al-dia/por-covid-19-endurecen-bloqueos-para-evitar-la-entrada-municipios-al-sur-del-edomex> |  |
| **Michoacán** | [**Suspensión clases a partir del 17.Marzo.2020**](https://michoacan.gob.mx/prensa/discursos/plan-de-acciones-extraordinarias-y-de-prevencion-contra-el-covid-19/) | [**DOF: Medidas preventivas COVID-19 (24.Marzo.2020)**](https://www.dof.gob.mx/nota_detalle.php?codigo=5590339&fecha=24/03/2020) | [**Plan de acciones extraordinarias y de prevención contra COVID-19 (16 Marzo 2020)**](https://michoacan.gob.mx/prensa/discursos/plan-de-acciones-extraordinarias-y-de-prevencion-contra-el-covid-19/) | [**Transporte NO suspende actividades (31 Marzo 2020)**](https://michoacan.gob.mx/prensa/discursos/suspende-gobierno-del-estado-actividades-no-esenciales-por-covid-19/) | [**Plan de acciones extraordinarias y de prevención contra COVID-19 (16 Marzo 2020)**](https://michoacan.gob.mx/prensa/discursos/plan-de-acciones-extraordinarias-y-de-prevencion-contra-el-covid-19/) | [**SSA: Preguntas frecuentes: ¿Existe restricción de viajes a nivel nacional? (12 Marzo 2020)**](https://www.gob.mx/salud/documentos/covid-19-preguntas-frecuentes?state=published) | [**México NO restringirá vuelos por COVID-19**](https://www.infobae.com/america/mexico/2020/03/12/mexico-no-restringira-vuelos-por-el-coronavirus-sct/) |
|  |  | [**DOF (31.03.20): Se extiende periodo de contingencia hasta el 30 Abril 2020.**](https://dof.gob.mx/nota_detalle.php?codigo=5590914&fecha=31/03/2020) | [**DOF (31.03.20): Eventos públicos de no más de 50 personas**](https://dof.gob.mx/nota_detalle.php?codigo=5590914&fecha=31/03/2020) | [**Disminuyen 40% del transporte público en Morelia (14 Abril 2020)**](https://www.elsoldemorelia.com.mx/local/detienen-40-por-ciento-de-las-unidades-del-transporte-por-covid-19-5096699.html) |  | [**Municipio de Michoacán cierra su entrada**](https://www.milenio.com/estados/mexico-215-municipios-cierran-entradas-temor-coronavirus) |  |
|  |  | [**DOF: Se extiende el periodo de contingencia hasta el 30 de Mayo 2020.**](https://www.dof.gob.mx/nota_detalle.php?codigo=5591876&fecha=17/04/2020) | [**DOF: Se extiende el periodo de contingencia hasta el 30 de Mayo 2020.**](https://www.dof.gob.mx/nota_detalle.php?codigo=5591876&fecha=17/04/2020) |  |  |  |  |
| **Morelos** | [**Suspende Morelos clases a partir del 18 Marzo 2020**](https://www.diariodemorelos.com/noticias/suspende-morelos-clases-y-eventos) | [**DOF: Medidas preventivas COVID-19 (24.Marzo.2020)**](https://www.dof.gob.mx/nota_detalle.php?codigo=5590339&fecha=24/03/2020) | [**Suspende Morelos eventos culturales a partir del 18 Marzo 2020**](https://www.diariodemorelos.com/noticias/suspende-morelos-clases-y-eventos) | [**DOF: Sistema de transporte NO se suspende (24.Marzo.20)**](https://www.dof.gob.mx/nota_detalle.php?codigo=5590339&fecha=24/03/2020) | [**Acciones preventivas ante presencia de COVID-19 (11 Marzo 2020)**](https://morelos.gob.mx/?q=prensa/nota/mantiene-secretaria-de-salud-acciones-preventivas-ante-presencia-de-coronavirus-covid-19) | [**SSA: Preguntas frecuentes: ¿Existe restricción de viajes a nivel nacional? (12 Marzo 2020)**](https://www.gob.mx/salud/documentos/covid-19-preguntas-frecuentes?state=published) | [**México NO restringirá vuelos por COVID-19**](https://www.infobae.com/america/mexico/2020/03/12/mexico-no-restringira-vuelos-por-el-coronavirus-sct/) |
|  |  | [**DOF (31.03.20): Se extiende periodo de contingencia hasta el 30 Abril 2020.**](https://dof.gob.mx/nota_detalle.php?codigo=5590914&fecha=31/03/2020) | [**DOF (31.03.20): Eventos públicos de no más de 50 personas**](https://dof.gob.mx/nota_detalle.php?codigo=5590914&fecha=31/03/2020) | [**Transporte público trabaja en un 50% (1 Abril 2020)**](https://www.elsoldecuernavaca.com.mx/finanzas/transporte-publico-trabaja-al-50-por-ciento-ante-coronavirus-en-morelos-5047596.html) |  | [**Municipios en Morelos cierran su entrada por temor a COVID**](https://www.diariodemorelos.com/noticias/cu-les-municipios-de-morelos-impiden-paso-de-turistas-por-temor-coronavirus) |  |
|  |  | [**DOF: Se extiende el periodo de contingencia hasta el 30 de Mayo 2020.**](https://www.dof.gob.mx/nota_detalle.php?codigo=5591876&fecha=17/04/2020) | [**DOF: Se extiende el periodo de contingencia hasta el 30 de Mayo 2020.**](https://www.dof.gob.mx/nota_detalle.php?codigo=5591876&fecha=17/04/2020) |  |  | [**Evitar viajes fuera del estado (17 Abril 2020)**](https://www.elsoldecuernavaca.com.mx/local/estas-son-las-nuevas-medidas-para-controlar-al-covid-19-en-cuernavaca-5116119.html) |  |
| **Nayarit** | [**Suspensión clases a partir del 20 Marzo 2020**](http://nayarit.gob.mx/notas/se_mantiene_nayarit_libre_de_coronavirus) | [**DOF: Medidas preventivas COVID-19 (24.Marzo.2020)**](https://www.dof.gob.mx/nota_detalle.php?codigo=5590339&fecha=24/03/2020) | [**Suspende Secretaría de Turismo eventos masivos (16 Marzo 2020)**](http://nayarit.gob.mx/notas/se_mantiene_nayarit_libre_de_coronavirus) | [**DOF: Sistema de transporte NO se suspende (24.Marzo.20)**](https://www.dof.gob.mx/nota_detalle.php?codigo=5590339&fecha=24/03/2020) | [**Decreto Estatal para Prevención y Detección COVID-19 (7 Marzo 2020)**](https://www.nayarit.gob.mx/docs/Decreto%20administrativo%20COVID-19.pdf) | [**SSA: Preguntas frecuentes: ¿Existe restricción de viajes a nivel nacional? (12 Marzo 2020)**](https://www.gob.mx/salud/documentos/covid-19-preguntas-frecuentes?state=published) | [**México NO restringirá vuelos por COVID-19**](https://www.infobae.com/america/mexico/2020/03/12/mexico-no-restringira-vuelos-por-el-coronavirus-sct/) |
|  |  | [**DOF (31.03.20): Se extiende periodo de contingencia hasta el 30 Abril 2020.**](https://dof.gob.mx/nota_detalle.php?codigo=5590914&fecha=31/03/2020) | [**DOF (31.03.20): Cancelacion de Eventos públicos**](https://dof.gob.mx/nota_detalle.php?codigo=5590914&fecha=31/03/2020) |  |  | [**Restricción entrada de playas (25 Marzo 2020)**](https://mexico.quadratin.com.mx/pide-secretaria-de-turismo-de-nayarit-no-visitar-sus-playas-por-pandemia/) | [**Secretaria Turismo cancela vuelos en Volaris de Tepic-Tijuana (16.Marzo.2020)**](http://nayarit.gob.mx/notas/se_mantiene_nayarit_libre_de_coronavirus) |
|  |  | [**DOF: Se extiende el periodo de contingencia hasta el 30 de Mayo 2020.**](https://www.dof.gob.mx/nota_detalle.php?codigo=5591876&fecha=17/04/2020) | [**DOF: Se extiende el periodo de contingencia hasta el 30 de Mayo 2020.**](https://www.dof.gob.mx/nota_detalle.php?codigo=5591876&fecha=17/04/2020) |  |  |  |  |
| **Nuevo Leon** | [**Mensaje del gobernador: Suspensión clases (17 Marzo 2020)**](http://www.nl.gob.mx/mensaje-del-gobernador-jaime-rodriguez-calderon-sobre-covid-19) | [**DOF: Medidas preventivas COVID-19 (24.Marzo.2020)**](https://www.dof.gob.mx/nota_detalle.php?codigo=5590339&fecha=24/03/2020) | [**Suspensión eventos masivos (14 Marzo 2020)**](http://nuevoleon.gob.mx/noticias/anuncia-estado-medidas-contra-covid-19) | [**DOF: Sistema de transporte NO se suspende (24.Marzo.20)**](https://www.dof.gob.mx/nota_detalle.php?codigo=5590339&fecha=24/03/2020) | [**Gráficos: Medidas de Prevención COVID-19 (18 Marzo 2020)**](http://www.nl.gob.mx/publicaciones/grafico-medidas-de-prevencion-contra-el-covid-19) | [**SSA: Preguntas frecuentes: ¿Existe restricción de viajes a nivel nacional? (12 Marzo 2020)**](https://www.gob.mx/salud/documentos/covid-19-preguntas-frecuentes?state=published) | [**México NO restringirá vuelos por COVID-19 (11 Marzo 2020)**](https://www.infobae.com/america/mexico/2020/03/12/mexico-no-restringira-vuelos-por-el-coronavirus-sct/) |
|  |  | [**DOF (31.03.20): Se extiende periodo de contingencia hasta el 30 Abril 2020.**](https://dof.gob.mx/nota_detalle.php?codigo=5590914&fecha=31/03/2020) | [**DOF (31.03.20): Cancelacion de Eventos públicos**](https://dof.gob.mx/nota_detalle.php?codigo=5590914&fecha=31/03/2020) | [**Restringen hora de transporte público en NL a partir del 27.04.20**](http://www.nl.gob.mx/noticias/restringiran-horarios-de-transporte-publico) |  | [**San Pedro Garza en NL implementa fase 4: cierra la entrada a su municipio**](https://www.forbes.com.mx/noticias-aplican-fase-4-por-covid-19-en-san-pedro-garza/) | [**Recomienda SSA no realizar viajes al extranjero (15 Marzo 2020)**](http://www.nl.gob.mx/noticias/suman-5-casos-de-covid-19-en-nuevo-leon) |
|  |  | [**DOF: Se extiende el periodo de contingencia hasta el 30 de Mayo 2020.**](https://www.dof.gob.mx/nota_detalle.php?codigo=5591876&fecha=17/04/2020) | [**DOF: Se extiende el periodo de contingencia hasta el 30 de Mayo 2020.**](https://www.dof.gob.mx/nota_detalle.php?codigo=5591876&fecha=17/04/2020) |  |  |  |  |
| **Oaxaca** | [**Suspende Oaxaca clases (18 marzo 2020)**](https://imparcialoaxaca.mx/oaxaca/417298/adelantan-escuelas-suspension-de-clases-en-la-capital/) | [**DOF: Medidas preventivas COVID-19 (24.Marzo.2020)**](https://www.dof.gob.mx/nota_detalle.php?codigo=5590339&fecha=24/03/2020) | [**Suspensión eventos culturales/artísticos en OAX (13 Marzo 2020)**](https://oaxaca.eluniversal.com.mx/estatal/13-03-2020/cancelan-dia-de-la-samaritana-y-otros-eventos-publicos-en-oaxaca-por-coronavirus) | [**DOF: Sistema de transporte NO se suspende (24.Marzo.20)**](https://www.dof.gob.mx/nota_detalle.php?codigo=5590339&fecha=24/03/2020) | [**Decreto para fortalecer medidas preventivas contra COVID-19(25 Marzo 2020)**](https://www.oaxaca.gob.mx/comunicacion/emite-amh-decreto-para-fortalecer-medidas-preventivas-contra-covid-19/) | [**Aeropuertos/Terminal de autobuses abiertas (2 Abril 2020)**](https://www.oaxaca.gob.mx/comunicacion/alejandro-murat-anuncia-medidas-de-seguridad-obligatorias-para-reducir-la-propagacion-del-covid-19/) | [**Evitar viajes NO esenciales internacionales**](https://www.oaxaca.gob.mx/salud/wp-content/uploads/sites/32/2020/02/UIES-OAXACA.-PREGUNTAS-Y-RESPUESTAS-COVID-19.pdf.pdf.pdf) |
|  |  | [**DOF (31.03.20): Se extiende periodo de contingencia hasta el 30 Abril 2020.**](https://dof.gob.mx/nota_detalle.php?codigo=5590914&fecha=31/03/2020) | [**DOF (31.03.20): Cancelacion de Eventos públicos**](https://dof.gob.mx/nota_detalle.php?codigo=5590914&fecha=31/03/2020) | [**No rebasar 50% de pasajeros en transporte público (31 Marzo 2020)**](https://www.oaxaca.gob.mx/comunicacion/policia-vial-y-semovi-refuerzan-acciones-para-evitar-el-sobrecupo-en-el-transporte-publico/) |  | [**Cierre temporal de playas (3 Abril 2020)**](https://www.oaxaca.gob.mx/wp-content/uploads/2020/04/EXT-COVID19GOB-2020-04-03.pdf) |  |
|  |  | [**DOF: Se extiende el periodo de contingencia hasta el 30 de Mayo 2020.**](https://www.dof.gob.mx/nota_detalle.php?codigo=5591876&fecha=17/04/2020) | [**DOF: Se extiende el periodo de contingencia hasta el 30 de Mayo 2020.**](https://www.dof.gob.mx/nota_detalle.php?codigo=5591876&fecha=17/04/2020) |  |  |  |  |
| **Puebla** | [**Puebla NO cancelará clases hasta que sea decreto federal**](http://sep.puebla.gob.mx/index.php/comunicados/la-secretaria-de-educacion-reitera-que-no-suspendera-las-clases-y-exhorta-a-todos-los-trabajadores-y-a-la-comunidad-estudiantil-a-mantener-las-medidas-de-prevencion) | [**DOF: Medidas preventivas COVID-19 (24.Marzo.2020)**](https://www.dof.gob.mx/nota_detalle.php?codigo=5590339&fecha=24/03/2020) | [**Suspensión de eventos culturas (23 Marzo 2020)**](https://www.puebla.gob.mx/index.php/noticias/item/1352-ordena-gobierno-del-estado-clausura-temporal-de-espacios-con-alta-concentracion-de-personas) | [**DOF: Sistema de transporte NO se suspende (24.Marzo.20)**](https://www.dof.gob.mx/nota_detalle.php?codigo=5590339&fecha=24/03/2020) | [**Medidas de prevención en aulas(03 Marzo 2020)**](http://sep.puebla.gob.mx/index.php/comunicados/sep-informa-medidas-de-prevencion-el-aulas) | [**Barbosa: No restringe las libertades de la sociedad (1 Abril 2020)**](https://www.puebla.gob.mx/index.php/noticias/item/1413-ante-contingencia-llama-barbosa-huerta-a-ediles-a-no-restringir-libertades-de-la-sociedad) | [**México NO restringirá vuelos por COVID-19 (11 Marzo 2020)**](https://www.infobae.com/america/mexico/2020/03/12/mexico-no-restringira-vuelos-por-el-coronavirus-sct/) |
|  | [**DOF: Suspensión clase (23 Marzo 2020)**](https://www.dof.gob.mx/nota_detalle.php?codigo=5589479&fecha=16/03/2020) | [**DOF (31.03.20): Se extiende periodo de contingencia hasta el 30 Abril 2020.**](https://dof.gob.mx/nota_detalle.php?codigo=5590914&fecha=31/03/2020) | [**DOF (31.03.20): Cancelacion de Eventos públicos**](https://dof.gob.mx/nota_detalle.php?codigo=5590914&fecha=31/03/2020) | [**Reduce un 20% el transporte público en Puebla por fase 3 (21 Abril 2020)**](https://www.elsoldepuebla.com.mx/local/reduciran-en-20-transporte-publico-en-puebla-capital-ante-fase-3-de-coronavirus-covid-19-5129736.html) |  | [**Municipios de Puebla restringen acceso a externos para prevenir contagios**](https://www.elsoldepuebla.com.mx/local/estado/con-filtros-y-cierres-buscan-frenar-covid-19-en-municipios-de-puebla-se-protegen-coronavirus-pandemia-emergencia-sanitaria-sanitizacion-5138756.html) | [**Barbosa: No restringe las libertades de la sociedad (1 Abril 2020)**](https://www.puebla.gob.mx/index.php/noticias/item/1413-ante-contingencia-llama-barbosa-huerta-a-ediles-a-no-restringir-libertades-de-la-sociedad) |
|  |  | [**DOF: Se extiende el periodo de contingencia hasta el 30 de Mayo 2020.**](https://www.dof.gob.mx/nota_detalle.php?codigo=5591876&fecha=17/04/2020) | [**DOF: Se extiende el periodo de contingencia hasta el 30 de Mayo 2020.**](https://www.dof.gob.mx/nota_detalle.php?codigo=5591876&fecha=17/04/2020) |  |  |  |  |
| **Queretaro** | [**Suspensión clases en QRO a partir del 18 Marzo 2020**](https://www.queretaro.gob.mx/documentos_interna_prensa.aspx?q=epsUWZ+4EQGJJB9Up0716g==) | [**DOF: Medidas preventivas COVID-19 (24.Marzo.2020)**](https://www.dof.gob.mx/nota_detalle.php?codigo=5590339&fecha=24/03/2020) | [**QRO: Suspende eventos masivos (14 Marzo 2020)**](https://www.eluniversalqueretaro.mx/politica/gobierno-de-queretaro-suspende-eventos-masivos-ante-covid-19) | [**DOF: Sistema de transporte NO se suspende (24.Marzo.20)**](https://www.dof.gob.mx/nota_detalle.php?codigo=5590339&fecha=24/03/2020) | [**Primer llamado de prevención ante COVID por parte del gobernador (12 Marzo 2020)**](https://www.queretaro.gob.mx/documentos_interna_prensa.aspx?q=epsUWZ+4EQE4oa+jX68hbg==) | [**DOF:Instituciones de transporte, aeropuertos siguen laborando**](https://www.dof.gob.mx/nota_detalle.php?codigo=5590339&fecha=24/03/2020) | [**México NO restringirá vuelos por COVID-19**](https://www.infobae.com/america/mexico/2020/03/12/mexico-no-restringira-vuelos-por-el-coronavirus-sct/) |
|  |  | [**DOF (31.03.20): Se extiende periodo de contingencia hasta el 30 Abril 2020.**](https://dof.gob.mx/nota_detalle.php?codigo=5590914&fecha=31/03/2020) | [**DOF (31.03.20): Cancelacion de Eventos públicos**](https://dof.gob.mx/nota_detalle.php?codigo=5590914&fecha=31/03/2020) | [**Uso de transporte público en Qro. disminuye por contingencia.**](https://www.eluniversalqueretaro.mx/metropoli/uso-de-transporte-publico-bajo-60-senala-el-itq) |  |  |  |
|  |  | [**DOF: Se extiende el periodo de contingencia hasta el 30 de Mayo 2020.**](https://www.dof.gob.mx/nota_detalle.php?codigo=5591876&fecha=17/04/2020) | [**DOF: Se extiende el periodo de contingencia hasta el 30 de Mayo 2020.**](https://www.dof.gob.mx/nota_detalle.php?codigo=5591876&fecha=17/04/2020) |  |  |  |  |
| **Quintana Roo** | [**DOF: Suspensión clase (23 Marzo 2020)**](https://www.dof.gob.mx/nota_detalle.php?codigo=5589479&fecha=16/03/2020) | [**DOF: Medidas preventivas COVID-19 (24.Marzo.2020)**](https://www.dof.gob.mx/nota_detalle.php?codigo=5590339&fecha=24/03/2020) | [**DOF (31.03.20): Cancelacion de Eventos públicos**](https://dof.gob.mx/nota_detalle.php?codigo=5590914&fecha=31/03/2020) | [**DOF: Sistema de transporte NO se suspende (24.Marzo.20)**](https://www.dof.gob.mx/nota_detalle.php?codigo=5590339&fecha=24/03/2020) | [**Comunicado Técnico: Medidas de prevención ante COVID de la Dirección General de Promoción en Salud**](https://cloud.salud.qroo.gob.mx/index.php/s/IuzpjjrEm3fokFq#pdfviewer) | [**No habrá restricciones en QRoo en cuestión de viajes por COVID-19**](https://qroo.gob.mx/portal/no-hay-restricciones-de-viaje-o-al-comercio-por-coronavirus-en-quintana-roo/) | [**No habrá restricciones en QRoo en cuestión de viajes por COVID-19**](https://qroo.gob.mx/portal/no-hay-restricciones-de-viaje-o-al-comercio-por-coronavirus-en-quintana-roo/) |
|  |  | [**DOF (31.03.20): Se extiende periodo de contingencia hasta el 30 Abril 2020.**](https://dof.gob.mx/nota_detalle.php?codigo=5590914&fecha=31/03/2020) | [**DOF: Se extiende el periodo de contingencia hasta el 30 de Mayo 2020.**](https://www.dof.gob.mx/nota_detalle.php?codigo=5591876&fecha=17/04/2020) | [**Suspensión de trasnporte público en COZUMEL (3 Abril 2020)**](https://quintanaroo.quadratin.com.mx/anuncia-pedro-joaquin-suspension-del-transporte-publico-en-cozumel/) |  | [**Cierran paso a Holbox para evitar contagios por COVID-19.**](https://www.eluniversal.com.mx/estados/acuerdan-pobladores-impedir-acceso-turistas-holbox-ante-epidemia) |  |
|  |  | [**DOF: Se extiende el periodo de contingencia hasta el 30 de Mayo 2020.**](https://www.dof.gob.mx/nota_detalle.php?codigo=5591876&fecha=17/04/2020) |  |  |  | [**Cierre Playas en Quintana Roo**](https://www.excelsior.com.mx/nacional/cierran-playas-en-quintana-roo-por-covid-19/1373823) |  |
| **San Luis Potosí** | <https://slp.gob.mx/SEGAM/Documentos%20compartidos/Suspensi%C3%B3n%20COVID19.pdf> | [**DOF: Medidas preventivas COVID-19 (24.Marzo.2020)**](https://www.dof.gob.mx/nota_detalle.php?codigo=5590339&fecha=24/03/2020) | <https://sanluis.eluniversal.com.mx/sociedad/16-03-2020/gobierno-de-slp-implementa-medidas-contra-el-coronavirus> | <https://www.elsoldesanluis.com.mx/local/slp-se-quedara-sin-transporte-publico-en-los-proximos-dias-5099648.html> | <https://slp.gob.mx/sitionuevo/Paginas/GaleriaVideos.aspx> |  |  |
|  |  | <https://sanluis.eluniversal.com.mx/sociedad/31-03-2020/coepris-supervisara-establecimientos-en-slp-durante-contingencia-por-coronavirus> |  |  | de acuerdo con indicador más antiuo |  |  |
| **Sinaloa** | <https://sinaloa.gob.mx/noticias/hagamos-de-la-prevencion-un-habito-frente-al-coronavirus-quirino#sthash.1BgAthJu.R2u3k4oQ.dpbs> | [**DOF: Medidas preventivas COVID-19 (24.Marzo.2020)**](https://www.dof.gob.mx/nota_detalle.php?codigo=5590339&fecha=24/03/2020) | <https://www.eluniversal.com.mx/estados/coronavirus-suspenden-eventos-masivos-en-sinaloa-y-san-luis-potosi> | <https://www.debate.com.mx/culiacan/Sacan-de-ruta-al-30-por-ciento-de-camiones-urbanos-en-Culiacan-20200324-0024.html> | <http://saludsinaloa.gob.mx/> | <https://www.debate.com.mx/guamuchil/Sinaloa-quedate-en-casa-Cierran-playas-de-Angostura-20200404-0275.html> |  |
|  |  | <https://twitter.com/Noticierista/status/1243320377494953994?s=20> | No especifica día, se toma de acuerdo a día de noticia |  | <https://sinaloa.gob.mx/noticias/jornadas-de-apoyo-operan-como-centros-de-valoracion-para-prevenir-y-detectar-el-coronavirus#sthash.4F4rfOCc.Tb1Cadhe.dpbs> |  |  |
| **Sonora** | <https://politica.expansion.mx/estados/2020/03/17/estados-adelantan-suspension-de-clases> | <https://twitter.com/gobiernosonora/status/1241777319154307074?s=21> | <https://opinionsonora.com/2020/03/16/cancelan-eventos-masivos-en-la-region-por-el-coronavirus/> | <https://www.elimparcial.com/sonora/hermosillo/Circularan-80-camiones-menos-en-Hermosillo-20200328-0002.html> | <https://www.radioformula.com.mx/noticias/20200401/ante-la-emergencia-sanitaria-todosjalamos-en-sonora-contra-el-covid-19/> | <https://www.debate.com.mx/losmochis/Cierran-las-playas-de-Sonora-por-coronavirus--20200320-0220.html> |  |
|  |  | [**DOF (31.03.20): Se extiende periodo de contingencia hasta el 30 Abril 2020.**](https://dof.gob.mx/nota_detalle.php?codigo=5590914&fecha=31/03/2020) |  |  | de acuerdo con indicador más antiguo |  |  |
| **Tabasco** | <https://www.dof.gob.mx/nota_detalle.php?codigo=5589479&fecha=16/03/2020> | <https://tabasco.gob.mx/PeriodicoOficial/descargar/1517> | <https://corat.mx/se-cancela-la-feria-tabasco-2020-y-eventos-masivos-anuncia-adan-augusto/> | <https://corat.mx/disminuye-demanda-en-el-servicio-de-transporte-publico-de-villahermosa/> | <https://tabasco.gob.mx/salud> | <https://tabasco.gob.mx/noticias/estamos-preparados-para-enfrentar-covid-19-adan-augusto> |  |
|  |  | [**DOF (31.03.20): Se extiende periodo de contingencia hasta el 30 Abril 2020.**](https://dof.gob.mx/nota_detalle.php?codigo=5590914&fecha=31/03/2020) |  |  | Se modificó de acuerdo a variable más antigua |  |  |
| **Tamaulipas** | <https://www.excelsior.com.mx/nacional/desde-hoy-suspenden-clases-en-10-estados-ante-pandemia/1370186> | [**DOF: Medidas preventivas COVID-19 (24.Marzo.2020)**](https://www.dof.gob.mx/nota_detalle.php?codigo=5590339&fecha=24/03/2020) | <https://www.milenio.com/ciencia-y-salud/coronavirus-cancelan-conciertos-zona-sur-tamaulipas> | <https://www.elsoldetampico.com.mx/local/suspenderan-un-50-del-servicio-de-transporte-publico-en-la-zona-5021510.html> | <http://coronavirus.tamaulipas.gob.mx/> | <https://www.tamaulipas.gob.mx/haciendoequipo/2020/03/23/conoce-que-medidas-esta-tomando-el-gobierno-de-tamaulipas-ante-el-coronavirus/> |  |
|  |  | <https://www.tamaulipas.gob.mx/salud/2020/03/mantener-la-sana-distancia-clave-para-disminuir-propagacion-del-covid-19/> | <https://www.tamaulipas.gob.mx/haciendoequipo/2020/03/23/conoce-que-medidas-esta-tomando-el-gobierno-de-tamaulipas-ante-el-coronavirus/> |  |  |  |  |
| **Tlaxcala** | <https://www.excelsior.com.mx/nacional/desde-hoy-suspenden-clases-en-10-estados-ante-pandemia/1370186> | <https://www.elsoldetlaxcala.com.mx/mexico/que-es-jornada-de-sana-distancia-coronavirus-covid-19-inicio-23-marzo-prevencion-suspension-temporal-actividades-no-esenciales-eventos-de-concentracion-masiva-4972716.html> | <https://twitter.com/gobtlaxcala/status/1244094488831619072?s=21> | <https://www.milenio.com/politica/comunidad/disminuye-90-afluencia-transporte-publico-tlaxcala> | [tlaxcala.gob.mx](http://tlaxcala.gob.mx/) |  |  |
|  |  | [**DOF (31.03.20): Se extiende periodo de contingencia hasta el 30 Abril 2020.**](https://dof.gob.mx/nota_detalle.php?codigo=5590914&fecha=31/03/2020) |  |  | De acuerdo a variable más antigua |  |  |
| **Veracruz** | <http://coronavirus.veracruz.gob.mx/2020/03/16/suspension-de-clases-en-veracruz-boca-del-rio-medellin-y-alvarado-a-partir-del-17-de-marzo-2/> | [**DOF: Medidas preventivas COVID-19 (24.Marzo.2020)**](https://www.dof.gob.mx/nota_detalle.php?codigo=5590339&fecha=24/03/2020) | <https://www.diariodexalapa.com.mx/local/cancelan-actos-en-veracruz-por-temos-al-coronavirus-veracruz-pandemia-covid-19-4969752.html> | <https://www.eldictamen.mx/veracruz/boca-ver/camiones-urbanos-anuncian-disminucion-de-corridas-por-covid-19/> | [coronavirus.veracruz.gob.mx](http://coronavirus.veracruz.gob.mx/) | <https://politica.expansion.mx/estados/2020/04/01/estado-por-estado-medidas-que-se-han-tomado-contra-covid-19> |  |
|  |  | <http://www.veracruz.gob.mx/2020/03/30/comunicado-estrategia-estatal-contra-el-coronavirus-30-03-2020/> |  |  | Comenzó con la estrategia vs Covid |  |  |
| **Yucatan** | <https://www.milenio.com/politica/jalisco-guanajuato-yucatan-adelantan-suspension-clases> | [**DOF: Medidas preventivas COVID-19 (24.Marzo.2020)**](https://www.dof.gob.mx/nota_detalle.php?codigo=5590339&fecha=24/03/2020) | ttps://www.yucatan.com.mx/merida/posponen-varios-eventos-masivos-en-merida | <https://laverdadnoticias.com/yucatan/Merida-anuncia-que-habra-menos-camiones-de-transporte-por-falta-de-gente-20200321-0145.html> | <https://twitter.com/salud_yucatan/status/1246100479471497216?s=21> | https://twitter.com/gobyucatan/status/1243574663088869384?s=21 https://www.razon.com.mx/estados/yucatan-playa-no-son-vacaciones/ | <https://www.jornada.com.mx/ultimas/estados/2020/03/14/cancelan-llegada-de-cruceros-a-yucatan-por-coronavirus-562.html> |
|  |  | [**DOF (31.03.20): Se extiende periodo de contingencia hasta el 30 Abril 2020.**](https://dof.gob.mx/nota_detalle.php?codigo=5590914&fecha=31/03/2020) | <https://www.latimes.com/espanol/mexico/articulo/2020-03-30/mexico-extiende-el-cierre-tras-aumentar-casos-de-covid-19-video> |  | De acuerdo a variable más antigua |  |  |
|  |  | <https://www.yucatan.com.mx/merida/ordenan-cierre-de-empresas-de-actividades-no-esenciales-en-yucatan> | <https://www.merida.gob.mx/capitalcultural/eventos.phpx> |  |  |  |  |
| **Zacatecas** | <https://www.elsoldezacatecas.com.mx/local/en-zacatecas-se-recorre-el-periodo-vacacional-de-semana-santa-coronavirus-covid-19-secretaria-de-educacion-reunion-aislamiento-voluntario-4971721.html> | [**DOF: Medidas preventivas COVID-19 (24.Marzo.2020)**](https://www.dof.gob.mx/nota_detalle.php?codigo=5590339&fecha=24/03/2020) | <https://www.eluniversal.com.mx/estados/coronavirus-suspenden-clases-en-universidad-y-cancelan-ferias-en-zacatecas> | <https://pagina24zacatecas.com.mx/2020/04/12/local/transporte-publico-resienten-efectos-negativos-por-la-contingencia-sanitaria/> | <https://twitter.com/si_sizart/status/1243931392955695105?s=21> |  |  |
|  |  | [**DOF (31.03.20): Se extiende periodo de contingencia hasta el 30 Abril 2020.**](https://dof.gob.mx/nota_detalle.php?codigo=5590914&fecha=31/03/2020) |  |  | De acuerdo a variable más antigua |  |  |

**Table 2a: Total Deaths, Mortality Rate, and Fatality Rate by State.**

|  |  |  |  |  |  |  |  |  |  |  |  |  |  |  |  |  |  |  |  |  |  |  |  |  |  |  |  |  |  |
| --- | --- | --- | --- | --- | --- | --- | --- | --- | --- | --- | --- | --- | --- | --- | --- | --- | --- | --- | --- | --- | --- | --- | --- | --- | --- | --- | --- | --- | --- |
|  | **Total Deaths** | | | | | | | | |  | **Mortality rate x 100,000 Residents** | | | | | | | | |  | **Fatality rate** | | | | | | | | |
|  | **Mar-31** | **Apr-30** | **May-31** | **Jun-30** | **Jul-31** | **Aug-31** | **Sep-30** | **Oct-31** | **Nov-30** |  | **Mar-31** | **Apr-30** | **May-31** | **Jun-30** | **Jul-31** | **Aug-31** | **Sep-30** | **Oct-31** | **Nov-30** |  | **Mar-31** | **Apr-30** | **May-31** | **Jun-30** | **Jul-31** | **Aug-31** | **Sep-30** | **Oct-31** | **Nov-30** |
|  |  |  |  |  |  |  |  |  |  |  |  |  |  |  |  |  |  |  |  |  |  |  |  |  |  |  |  |  |  |
| **Aguascalientes** | 0 | 6 | 35 | 146 | 260 | 405 | 652 | 851 | 1,114 |  | 0.00 | 0.42 | 2.44 | 10.18 | 18.12 | 28.23 | 45.45 | 59.32 | 77.65 |  | 0.00 | 0.03 | 0.04 | 0.06 | 0.06 | 0.07 | 0.09 | 0.09 | 0.08 |
| **Baja California** | 0 | 222 | 865 | 1,931 | 2,604 | 3,145 | 3,526 | 3,838 | 4,281 |  | 0.00 | 6.11 | 23.80 | 53.12 | 71.64 | 86.52 | 97.00 | 105.59 | 117.78 |  | 0.00 | 0.14 | 0.17 | 0.22 | 0.19 | 0.19 | 0.18 | 0.17 | 0.16 |
| **Baja California Sur** | 0 | 16 | 35 | 71 | 174 | 354 | 462 | 608 | 665 |  | 0.00 | 1.99 | 4.35 | 8.82 | 21.62 | 43.99 | 57.41 | 75.56 | 82.64 |  | 0.00 | 0.05 | 0.06 | 0.05 | 0.04 | 0.05 | 0.05 | 0.05 | 0.05 |
| **Campeche** | 0 | 19 | 90 | 205 | 482 | 755 | 825 | 862 | 926 |  | 0.00 | 1.90 | 8.99 | 20.49 | 48.17 | 75.45 | 82.45 | 86.15 | 92.54 |  | 0.00 | 0.16 | 0.15 | 0.11 | 0.11 | 0.14 | 0.14 | 0.13 | 0.14 |
| **Coahuila** | 2 | 41 | 81 | 229 | 623 | 1,379 | 1,883 | 2,428 | 3,329 |  | 0.06 | 1.27 | 2.52 | 7.11 | 19.36 | 42.84 | 58.50 | 75.43 | 103.43 |  | 0.05 | 0.10 | 0.07 | 0.05 | 0.05 | 0.06 | 0.07 | 0.07 | 0.08 |
| **Colima** | 0 | 3 | 21 | 71 | 206 | 429 | 568 | 744 | 804 |  | 0.00 | 0.38 | 2.67 | 9.04 | 26.24 | 54.64 | 72.34 | 94.76 | 102.40 |  | 0.00 | 0.11 | 0.14 | 0.13 | 0.11 | 0.12 | 0.12 | 0.11 | 0.11 |
| **Chiapas** | 0 | 7 | 135 | 543 | 898 | 1,001 | 1,020 | 1,069 | 1,089 |  | 0.00 | 0.12 | 2.36 | 9.48 | 15.67 | 17.47 | 17.80 | 18.66 | 19.00 |  | 0.00 | 0.04 | 0.07 | 0.12 | 0.16 | 0.16 | 0.16 | 0.14 | 0.14 |
| **Chihuahua** | 0 | 76 | 318 | 640 | 888 | 1,124 | 1,382 | 1,993 | 3,572 |  | 0.00 | 2.00 | 8.37 | 16.84 | 23.36 | 29.57 | 36.35 | 52.43 | 93.96 |  | 0.00 | 0.22 | 0.20 | 0.21 | 0.17 | 0.15 | 0.12 | 0.11 | 0.12 |
| **Mexico City** | 8 | 409 | 2,658 | 6,560 | 8,920 | 10,532 | 12,121 | 15,173 | 17,581 |  | 0.09 | 4.52 | 29.36 | 72.45 | 98.52 | 116.32 | 133.87 | 167.58 | 194.18 |  | 0.04 | 0.07 | 0.11 | 0.14 | 0.12 | 0.11 | 0.10 | 0.09 | 0.08 |
| **Durango** | 1 | 7 | 37 | 142 | 276 | 440 | 623 | 803 | 1,121 |  | 0.05 | 0.37 | 1.98 | 7.60 | 14.77 | 23.54 | 33.33 | 42.96 | 59.98 |  | 0.14 | 0.11 | 0.10 | 0.07 | 0.07 | 0.07 | 0.07 | 0.06 | 0.05 |
| **Guanajuato** | 0 | 24 | 107 | 357 | 971 | 2,041 | 2,907 | 3,450 | 3,975 |  | 0.00 | 0.39 | 1.72 | 5.73 | 15.59 | 32.77 | 46.67 | 55.39 | 63.82 |  | 0.00 | 0.09 | 0.07 | 0.05 | 0.05 | 0.06 | 0.07 | 0.07 | 0.06 |
| **Guerrero** | 0 | 47 | 266 | 880 | 1,380 | 1,658 | 1,886 | 2,174 | 2,379 |  | 0.00 | 1.29 | 7.27 | 24.06 | 37.74 | 45.34 | 51.57 | 59.45 | 65.05 |  | 0.00 | 0.17 | 0.14 | 0.16 | 0.13 | 0.11 | 0.10 | 0.10 | 0.10 |
| **Hidalgo** | 3 | 30 | 304 | 666 | 1,059 | 1,607 | 1,981 | 2,286 | 2,693 |  | 0.10 | 0.97 | 9.85 | 21.58 | 34.31 | 52.07 | 64.18 | 74.07 | 87.25 |  | 0.16 | 0.11 | 0.18 | 0.17 | 0.16 | 0.16 | 0.16 | 0.14 | 0.14 |
| **Jalisco** | 3 | 28 | 143 | 692 | 1,524 | 2,428 | 3,295 | 4,063 | 4,766 |  | 0.04 | 0.33 | 1.70 | 8.23 | 18.12 | 28.87 | 39.18 | 48.31 | 56.67 |  | 0.03 | 0.08 | 0.08 | 0.10 | 0.12 | 0.12 | 0.12 | 0.12 | 0.12 |
| **State of Mexico** | 1 | 169 | 1,159 | 4,312 | 6,379 | 8,017 | 9,442 | 10,704 | 11,914 |  | 0.01 | 0.97 | 6.65 | 24.74 | 36.60 | 46.00 | 54.18 | 61.42 | 68.36 |  | 0.01 | 0.05 | 0.08 | 0.13 | 0.12 | 0.12 | 0.12 | 0.11 | 0.11 |
| **Michoacan** | 1 | 42 | 165 | 450 | 755 | 1,157 | 1,627 | 1,997 | 2,284 |  | 0.02 | 0.87 | 3.42 | 9.33 | 15.65 | 23.98 | 33.72 | 41.39 | 47.33 |  | 0.05 | 0.13 | 0.09 | 0.08 | 0.08 | 0.08 | 0.08 | 0.08 | 0.08 |
| **Morelos** | 2 | 43 | 265 | 665 | 839 | 967 | 1,097 | 1,191 | 1,288 |  | 0.10 | 2.10 | 12.96 | 32.53 | 41.05 | 47.31 | 53.67 | 58.27 | 63.01 |  | 0.29 | 0.13 | 0.19 | 0.23 | 0.21 | 0.19 | 0.18 | 0.17 | 0.16 |
| **Nayarit** | 0 | 12 | 53 | 201 | 387 | 579 | 734 | 867 | 980 |  | 0.00 | 0.93 | 4.11 | 15.60 | 30.03 | 44.93 | 56.96 | 67.28 | 76.05 |  | 0.00 | 0.13 | 0.09 | 0.12 | 0.11 | 0.12 | 0.12 | 0.13 | 0.14 |
| **Nuevo Leon** | 0 | 16 | 88 | 332 | 1,066 | 2,233 | 3,083 | 3,898 | 4,561 |  | 0.00 | 0.29 | 1.57 | 5.92 | 19.00 | 39.80 | 54.95 | 69.48 | 81.30 |  | 0.00 | 0.05 | 0.06 | 0.06 | 0.06 | 0.08 | 0.08 | 0.07 | 0.07 |
| **Oaxaca** | 1 | 19 | 152 | 591 | 934 | 1,246 | 1,405 | 1,645 | 1,851 |  | 0.02 | 0.46 | 3.67 | 14.26 | 22.54 | 30.07 | 33.91 | 39.70 | 44.67 |  | 0.07 | 0.14 | 0.11 | 0.11 | 0.09 | 0.09 | 0.08 | 0.08 | 0.08 |
| **Puebla** | 1 | 86 | 350 | 1,274 | 2,443 | 3,518 | 4,073 | 4,777 | 5,167 |  | 0.01 | 1.25 | 5.08 | 18.49 | 35.46 | 51.06 | 59.12 | 69.34 | 75.00 |  | 0.01 | 0.13 | 0.11 | 0.12 | 0.12 | 0.13 | 0.13 | 0.13 | 0.13 |
| **Querétaro** | 1 | 13 | 105 | 283 | 477 | 721 | 992 | 1,221 | 1,504 |  | 0.04 | 0.57 | 4.61 | 12.41 | 20.92 | 31.63 | 43.52 | 53.56 | 65.98 |  | 0.03 | 0.10 | 0.11 | 0.14 | 0.13 | 0.11 | 0.11 | 0.09 | 0.07 |
| **Quintana Roo** | 1 | 114 | 352 | 575 | 989 | 1,389 | 1,644 | 1,829 | 1,926 |  | 0.06 | 6.62 | 20.43 | 33.37 | 57.39 | 80.60 | 95.40 | 106.14 | 111.77 |  | 0.02 | 0.14 | 0.19 | 0.16 | 0.13 | 0.13 | 0.14 | 0.14 | 0.13 |
| **San Luis Potosi** | 2 | 7 | 51 | 154 | 486 | 1,167 | 1,690 | 2,079 | 2,492 |  | 0.07 | 0.24 | 1.78 | 5.37 | 16.96 | 40.72 | 58.96 | 72.54 | 86.95 |  | 0.08 | 0.07 | 0.05 | 0.05 | 0.05 | 0.06 | 0.07 | 0.07 | 0.07 |
| **Sinaloa** | 3 | 139 | 499 | 1,298 | 2,191 | 2,758 | 3,194 | 3,607 | 3,972 |  | 0.10 | 4.40 | 15.81 | 41.12 | 69.41 | 87.37 | 101.18 | 114.27 | 125.83 |  | 0.11 | 0.16 | 0.15 | 0.16 | 0.17 | 0.17 | 0.17 | 0.16 | 0.16 |
| **Sonora** | 0 | 20 | 111 | 708 | 1,951 | 2,646 | 2,899 | 3,170 | 3,464 |  | 0.00 | 0.65 | 3.61 | 23.03 | 63.45 | 86.06 | 94.28 | 103.10 | 112.66 |  | 0.00 | 0.09 | 0.05 | 0.09 | 0.11 | 0.12 | 0.12 | 0.08 | 0.08 |
| **Tabasco** | 0 | 124 | 520 | 1,048 | 1,988 | 2,565 | 2,827 | 3,039 | 3,134 |  | 0.00 | 4.82 | 20.22 | 40.74 | 77.29 | 99.72 | 109.90 | 118.14 | 121.84 |  | 0.00 | 0.13 | 0.13 | 0.10 | 0.09 | 0.09 | 0.09 | 0.09 | 0.08 |
| **Tamaulipas** | 0 | 18 | 108 | 363 | 1,013 | 1,793 | 2,247 | 2,674 | 3,000 |  | 0.00 | 0.49 | 2.96 | 9.94 | 27.75 | 49.12 | 61.55 | 73.25 | 82.18 |  | 0.00 | 0.06 | 0.07 | 0.06 | 0.06 | 0.07 | 0.08 | 0.08 | 0.08 |
| **Tlaxcala** | 0 | 23 | 148 | 335 | 673 | 900 | 1,027 | 1,146 | 1,204 |  | 0.00 | 1.67 | 10.72 | 24.28 | 48.77 | 65.22 | 74.42 | 83.04 | 87.25 |  | 0.00 | 0.12 | 0.14 | 0.13 | 0.15 | 0.14 | 0.14 | 0.14 | 0.13 |
| **Veracruz** | 1 | 52 | 518 | 1,547 | 2,755 | 3,666 | 4,222 | 4,838 | 5,539 |  | 0.01 | 0.61 | 6.07 | 18.12 | 32.26 | 42.93 | 49.44 | 56.65 | 64.86 |  | 0.04 | 0.09 | 0.14 | 0.15 | 0.13 | 0.13 | 0.13 | 0.13 | 0.14 |
| **Yucatan** | 0 | 26 | 155 | 414 | 874 | 1,314 | 1,606 | 1,789 | 2,051 |  | 0.00 | 1.15 | 6.86 | 18.33 | 38.69 | 58.16 | 71.09 | 79.19 | 90.79 |  | 0.00 | 0.06 | 0.09 | 0.09 | 0.09 | 0.09 | 0.09 | 0.08 | 0.09 |
| **Zacatecas** | 1 | 8 | 34 | 96 | 243 | 483 | 708 | 940 | 1,314 |  | 0.06 | 0.48 | 2.04 | 5.76 | 14.58 | 28.98 | 42.49 | 56.41 | 78.85 |  | 0.17 | 0.10 | 0.12 | 0.11 | 0.09 | 0.09 | 0.10 | 0.08 | 0.08 |
| **National** | **32** | **1,866** | **9,928** | **27,779** | **46,708** | **64,417** | **77,648** | **91,753** | **105,940** |  | **0.03** |  |  |  |  |  |  |  |  |  |  |  |  |  |  |  |  |  |  |
|  |  |  |  |  |  |  |  |  |  |  |  |  |  |  |  |  |  |  |  |  |  |  |  |  |  |  |  |  |  |
| Source: Our own estimates based on data from the Mexican Secretary of Health's General Epidemiological Directorate (Coronavirus COVID19 Comunicado Técnico Diario. Secretaría de Salud, México). https://www.gob.mx/salud/documentos/coronavirus-covid-19-comunicado-tecnico-diario-238449 | | | | | | | | | | | | | | | | | | | | | | | | | | | | | |

| **Table 3a. Correlation Matrix between Lagged Public Policy Index and Mortality in Mexico** | | | | | | | | |
| --- | --- | --- | --- | --- | --- | --- | --- | --- |
| **From February 27 to November 30 2020** | | | | | | | | |
|  |  |  |  |  |  |  |  |  |
|  |  | Policy index | Policy index (Lagged 2 weeks) | Total Deaths | Total Deaths per 100k | Fatality rate | Total Deaths (lagged 2 weeks) | Total Deaths Per 100k (lagged 2 weeks) |
| Policy index (Lagged 2 weeks) | Coef. Corr. | 0.89 |  |  |  |  |  |  |
|  | Stat. Sig. | 0.00 |  |  |  |  |  |  |
| Total Deaths | Coef. Corr. | 0.15 | 0.18 |  |  |  |  |  |
|  | Stat. Sig. | 0.00 | 0.00 |  |  |  |  |  |
| Total Deaths per 100k | Coef. Corr. | 0.19 | 0.25 | 0.69 |  |  |  |  |
|  | Stat. Sig. | 0.00 | 0.00 | 0.00 |  |  |  |  |
| Fatality rate | Coef. Corr. | 0.29 | 0.40 | 0.14 | 0.23 |  |  |  |
|  | Stat. Sig. | 0.00 | 0.00 | 0.00 | 0.00 |  |  |  |
| Total Deaths (lagged 2 weeks) | Coef. Corr. | 0.05 | 0.15 | 1.00 | 0.69 | 0.15 |  |  |
|  | Stat. Sig. | 0.00 | 0.00 | 0.00 | 0.00 | 0.00 |  |  |
| Total Deaths Per 100k (lagged 2 weeks) | Coef. Corr. | 0.03 | 0.20 | 0.68 | 0.99 | 0.24 | 0.70 |  |
|  | Stat. Sig. | 0.01 | 0.00 | 0.00 | 0.00 | 0.00 | 0.00 |  |
| Fatality rate (lagged 2 weeks) | Coef. Corr. | 0.16 | 0.28 | 0.15 | 0.25 | 0.68 | 0.17 | 0.27 |
|  | Stat. Sig. | 0.00 | 0.00 | 0.00 | 0.00 | 0.00 | 0.00 | 0.00 |
